# Determining the N-Glycan and Collagen/Extracellular Matrix Protein Compositions in a Novel Outcome Cohort of Prostate Cancer Tissue Microarrays Using MALDI-MSI

**DOI:** 10.1158/2767-9764.CRC-24-0152

**Published:** 2024-11-27

**Authors:** Jordan P. Hartig, Kaitlyn Bejar, Lyndsay E.A. Young, Grace Grimsley, Jennifer R. Bethard, Dean A. Troyer, Javier Hernandez, Jennifer D. Wu, Joseph E. Ippolito, Lauren E. Ball, Jonathan A.L. Gelfond, Teresa L. Johnson-Pais, Anand S. Mehta, Robin J. Leach, Peggi M. Angel, Richard R. Drake

**Affiliations:** 1 Medical University of South Carolina, Charleston, South Carolina.; 2 University of Texas Health Science Center, San Antonio, Texas.; 3 Eastern Virginia Medical School, Norfolk, Virginia.; 4 Audie L. Murphy Memorial Veteran’s Administration Hospital, San Antonio, Texas.; 5 Northwestern Feinberg School of Medicine, Chicago, Illinois.; 6 Washington University in St. Louis School of Medicine, St. Louis, Missouri.

## Abstract

Prostate cancer is a significant health concern, with metastasis posing major clinical challenges and resulting in poor patient outcome. Despite screening and treatment advances, a critical need for novel biomarkers to predict prostate cancer progression at the time of prostatectomy persists. Here, we assessed aberrant N-glycosylation patterns and alterations in extracellular matrix (ECM) proteins as potential biomarkers of predicting prostate cancer severity in a unique patient outcome cohort. Tissue microarray slides were assembled from primary prostatectomy specimens that were categorized into “no evidence of disease (NED)” and “metastasis (MET)” designations based on >5-year disease progression outcomes. Serial mass spectrometry imaging techniques were performed to analyze N-glycans and ECM components in formalin-fixed paraffin-embedded cores. The results revealed a significant upregulation of bisecting and multiantennary core-fucosylated N-glycans in MET tissues when compared with NED tissues. Alterations in ECM composition in both NED and MET cohorts were observed, particularly in collagen species and the amount of hydroxyproline content. Results suggest a coordinated alteration of ECM protein and glycosylation content in prostate cancer tissues can be predictive for postprostatectomy disease progression.

**Significance:**

Using matrix-assisted laser desorption/ionization mass spectrometry imaging techniques on a unique cohort of prostate cancer tissues, we highlighted several molecular characteristics of matrix that have potential to act as early predictors of prostate cancer metastasis.

## Introduction

Prostate cancer is the most diagnosed noncutaneous malignancy among American men. Although those men with localized and regional prostate cancer have a 5-year survival rate of 100%, patients with distant metastases have a 5-year survival rate of 32% (1). Roughly 20% to 40% of patients with prostate cancer undergoing radical prostatectomy and 30% to 50% of patients undergoing radiotherapy experience recurrence within 10 years (2–4). Patients with recurrence are usually treated with androgen deprivation therapy, and prolonged treatment can lead to the development of castration-resistant prostate cancer. In 25% to 30% of those with castration-resistant prostate cancer, treatment-induced neuroendocrine prostate cancer develops, which is a more lethal variant of prostate cancer , with an average survival time of <1 year (5–8). Multiple genomic classifier assays have demonstrated success in predicting biochemical recurrence and metastasis by analyzing RNA expression of key genes in biopsy or prostatectomy tissue, yielding AUC values for these classifiers between 0.70 and 0.80 (9). Combined with the overall low specificity for PSA screening (10), there remains an urgent need for novel clinical biomarkers that can predict the severity of prostate cancer at early stages.

One hallmark of prostate cancer incidence and progression is aberrant glycosylation (11, 12). N-glycosylation, in which an oligosaccharide is attached to asparagine on carrier glycoproteins, is a common cell surface posttranslational modification important in protein folding, cell signaling, tissue integrity, and other crucial cell–cell and cell–extracellular matrix (ECM) interactions. Under cancerous conditions, N-glycans are responsible for assisting in tumor cell invasion, immune modulation, angiogenesis, and metastasis (13, 14). The addition of one or more fucose sugars to N-glycans, termed fucosylation, is performed by a gene family of fucosyltransferases known to regulate cancer cell proliferation, invasiveness, and resistance to treatment (15, 16). Increased expression of fucosyltranserase 8 , the enzyme responsible for core fucosylation of N-glycans, has been directly related to an increase in invasion and metastasis for patients of several types of cancer, including non–small cell lung cancer, melanoma, and prostate cancer (15–17).The effects of aberrant N-glycosylation largely occur in the ECM, as well as an increase in the number of reactive stroma, which has also been associated with prostate cancer severity. A “reactive stroma” can be characterized by the remodeling of the ECM, including degradation of collagen type proteins, aberrant changes in growth factors and cytokines, and immune recruitment (18–20). This alteration is well classified in prostate cancer. When an epithelial tumor invades the surrounding connective tissue, a wound healing–like response is triggered by the host, leading to altered stromal architecture (19). A recent study reported that collagen is one of the key components of remodeling the microenvironment of the connective tissue in order to allow for prostate cancer progression (21). However, these changes have been poorly explored as predictors of prostate cancer severity.

Prior studies from our group have focused on characterizing the landscape of changes in N-glycosylation (22–26) and ECM (27) in clinical formalin-fixed, paraffin-embedded (FFPE) prostate cancer tissues obtained from pathology using matrix-assisted laser desorption/ionization mass spectrometry imaging (MALDI-MSI) technologies. The N-glycan MALDI-MSI studies involve the spraying of peptide N-glycosidase F onto the tissues to release the N-glycans, followed by MALDI-MSI analysis to localize them. The ECM MALDI-MSI analysis of prostate cancer tissues is done following spraying of a bacterial collagenase enzyme onto the tissue, followed by MALDI-MSI detection of cleaved ECM peptides (27). These tissue analysis workflows have not been applied to evaluate prostate cancer in the context of clinical outcomes and the ability to predict metastatic potential at the time of prostatectomy. A unique cohort of prostate cancer tissues representing primary prostatectomy samples was identified and linked to clinical outcome data greater than 5 years. Subjects were identified that had no evidence of disease progression, compared with others who had eventually progressed to metastatic prostate cancer. Tissue cores were assembled in five tissue microarray (TMA) slides and used in multiple sequential MALDI-MSI analyses of N-glycans and ECM proteins. An emphasis on core fucosylation differences in the detected N-glycans and hydroxyproline content of the ECM peptides was evaluated with the clinical data and patient outcome data. Multiple biomarker candidates to distinguish metastatic progression potential in prostate cancer tissues at the time of prostatectomy were identified.

## Materials and Methods

### TMA cohorts

A set of TMAs were constructed using 124 specimens from patients receiving prostatectomies as their primary treatment for their prostate cancer. Following prostatectomy, tissue samples were banked for a minimum of 5 years and subsequently categorized for the long-term outcome. The specimens were obtained from the Audie L. Murphy Memorial Veteran’s Administration (VA) Hospital through a joint institutional review board between UT Health San Antonio and the VA. The VA system utilizes a single electronic medical record platform, thus allowing the determination of long-term outcomes for each patient as long as they continued receiving care in the VA system. The outcomes were all confirmed by a board-certified urologic oncologist (J. Hernandez). Patients who had no evidence of recurrence of Institutional Review Board following initial prostatectomy were placed in the “no evidence of disease (i.e., no recurrence; NED)” cohort, whereas patients who had documented metastasis of prostate cancer following initial prostatectomy were placed in the “metastasis (MET)” cohort. Slides were prepared from stored tissue blocks and the prostate tumors were marked by a genitourinary pathologist (D.A. Troyer). TMAs were constructed by sampling 0.4 cm diameter tumor cores from these selected prostate cancer tissues. Because these are discovery TMAs, the samples were segregated by long-term outcome with adjacent tumor tissue specimens present on each slide. A summary of the quantity of tumor specimens in the TMAs, along with corresponding clinical and demographic data, is summarized in Table 1.

**Table 1:** Clinical characteristics for each patient in the cohort. There were 51 patients in the “MET” cohort and 73 patients in the “NED” cohort. Data from each patient were collected including age at time of prostatectomy, years of follow up, PSA levels at time of biopsy, race, lymph node status, and tumor margins. Tumor stage and Gleason score information are provided in Supplementary Table S4

Characteristic	MET, *N* = 51^a^	NED, *N* = 73^a^	*P*-value^b^
Age at RRP			
Median	64 (58, 67)	60 (57, 67)	0.2
Unknown	4	0	
Years of follow-up			
Median	5.5 (2.0, 9.0)	10.0 (6.0, 13.0)	<0.0001
Unknown	3	0	
PSA at RRP			
Median	9 (7, 21)	5 (4, 7)	<0.0001
Unknown	9	2	
PSA at biopsy			
Median	11 (7, 22)	5 (4, 7)	<0.0001
Unknown	6	6	
Race			
Asian	0 (0%)	1 (1.4%)	
Black	4 (8.3%)	27 (39%)	
White	44 (92%)	42 (60%)	
Unknown	3	3	
Lymph nodes			<0.001
Negative	24 (53%)	72 (100%)	
Positive	21 (47%)	0 (0%)	
Unknown	6	1	
Margins			0.027
Negative	23 (51%)	52 (71%)	
Positive	22 (49%)	21 (29%)	
Unknown	6	0	

Abbreviations: RRP, radical retropubic prostatectomy; Bx, biopsy.

a Median (IQR): *n* (%).

b Wilcoxon rank sum test: Fisher’s exact test: Pearson’s *χ*^2^ test.

### Materials

Chemicals and solvents (analytical grade) were purchased from the following sources: high-performance liquid chromatography grade acetonitrile (ACN), ethanol, xylene, water, DMSO, and ammonia were obtained from Thermo Fisher Scientific. LC/MS grade ACN, trifluoroacetic acid (TFA), and water were also obtained by Thermo Fischer Scientific. Citraconic anhydride was obtained from Thermo Fisher Scientific. Alpha-cyano-4-hydroxycinnamic acid (CHCA), sodium bicarbonate, Trizma base, 1-hydroxybenzotriazole hydrate, dimethylamine, propargylamine, and TFA were obtained from Sigma-Aldrich. Recombinant peptide N-glycosidase F (PNGase F) PRIME and endoglycosidase F3 (EndoF3) PRIME were obtained from N-Zyme Scientifics. Hematoxylin and eosin (H&E) stains were obtained from Cancer Diagnostics. Collagenase type III (COLaseIII; *Clostridium histolyticum*) was purchased from Worthington Biochemicals.

### N-glycan MALDI-MSI of FFPE tissue slides

Tissue preparation was performed according to previously established methods (28) and standardized protocols (29). Briefly, tissue slides were dewaxed and rehydrated before undergoing antigen retrieval in citraconic buffer (pH 3) for 30 minutes in a vegetable steamer at 100°C. Following buffer exchange and drying in a desiccator, 15 passes of PNGaseF enzyme at 0.1 mg/mL were applied as a dry molecular coating to the tissue slides at a rate of 25 mL/minutes with a velocity of 1,200 mm/minutes and a 3-mm offset at 10 psi and 45°C. Enzyme was applied with an M3 Sprayer (HTX Technologies). Following PNGaseF application, slides were incubated in prewarmed humidity chambers for 2 hours at 37°C for enzymatic cleavage of N-glycans. Following digestion, 7 mg/mL CHCA matrix in 50% ACN/0.1% TFA was applied by M3 Sprayer to slides at a rate of 100 mL/minutes with a velocity of 1,300 mm/minutes and a 2.5-mm offset at 10 psi and 79°C. Slides were desiccated after matrix application until analysis.

Imaging was performed on a timsTOF Flex MALDI-QTOF mass spectrometer (Bruker Corporation) as previously described (30). Data were collected in positive ion mode at mass ranges of 700 to 4,000 *m*/*z* with an 80-mm raster, 300 laser shots per pixel, and a laser spot size of 20 mm. Following acquisition, spectra were imported to SCiLS Lab software (Bruker Corporation) for processing. Spectra were normalized to total ion count and annotated based on an established in-house N-glycan database (30, 31) based off of prior characterizations of N-glycan structures via reversed-phase liquid chromatography–coupled tandem mass spectrometry, MALDI-TOF-MS/MS, and alternate enzymatic release experiments (30). A peak list of detected N-glycans is provided in Supplementary Table S1.

### Preparation and application of EndoF3 enzyme

EndoF3 enzyme was dialyzed before application to tissue samples. First, a 10 mmol/L stock solution of ammonium bicarbonate in high-performance liquid chromatography water was prepared. To 50 mL of stock solution, 100 mL of 500 mmol/L CaCl_2_ was added. Ammonium bicarbonate buffer was added to the dialysis tube and used to saturate the dialysis tube filter. Then, enzyme was pipetted on top of the filter, and the tube was placed on orbital for 2 hours. After 2 hours, the lower tube was emptied and filled with fresh ammonium bicarbonate/CaCl_2_ buffer. The tube was placed on orbital a second time for 3.5 hours. After 3.5 hours, EndoF3 was stored in a microtube at −20°C until application. At the time of application, enzyme was diluted to a total volume of 1 mL with fresh ammonium bicarbonate buffer and applied to tissue under the same application, incubation, and CHCA matrix application procedure as described for PNGaseF (17).

### Collagen/ECM MALDI-MSI of FFPE tissue slides

Tissue preparation was performed according to previously established methods (27, 32). Briefly, the EndoF3/PNGase tissue slides underwent a series of ethanol cleaning steps to remove the CHCA matrix, followed by H&E staining as previously described (27). High-resolution images of stained slides were collected on a Hamamatsu NanoZoomer 2.0RS. Tissue slides underwent antigen retrieval in tris buffer (pH 9) for 30 minutes in a vegetable steamer at 100°C. Following buffer exchange and drying in a desiccator, 15 passes of COLaseIII enzyme at 0.07 mg/mL were applied as a dry molecular coating at a rate of 25 mL/minutes with a velocity of 1,200 mm/minutes and a 3-mm offset at 10 psi and 45°C. Enzyme was applied with an M3 Sprayer (HTX Technologies), and slides were incubated in prewarmed humidity chambers for 5 hours at 37°C for enzymatic cleavage of collagen and ECM peptides. Following digestion, 7 mg/mL CHCA matrix in 50% ACN/1.0% TFA was applied by M3 Sprayer to slides at a rate of 100 mL/minutes with a velocity of 1,300 mm/minutes at a 2.5-mm offset at 10 psi and 79°C. Slides were dipped in ammonium phosphate buffer and desiccated until analysis.

Imaging was performed on a timsTOF Flex MALDI-QTOF mass spectrometer (Bruker Corporation) as previously described (27, 32, 33) Data were collected in positive ion mode at mass ranges 400 to 2,500 *m*/*z* with an 80-mm raster, 300 laser shots per pixel, and a laser spot size of 20 mm. Following acquisition, peptide spectra were imported to SCiLS Lab software (Bruker Corporation) for processing. Spectra were normalized to total ion count and annotated based on subsequently collected data based on detected proteins found via reversed-phase liquid chromatography–coupled tandem mass spectrometry analysis of homogenized tissue samples. A peak list of COLaseIII-digested peptides is provided in Supplementary Table S2.

### Proteomic confirmation of imaged peaks

Matrix was removed from slides following collagen imaging as described (27), and 10 cores were scraped off 1 slide from each cohort (20 cores total) into microcentrifuge tubes. Tissues were digested with COLaseIII (2 mg of enzyme per tissue section) at pH 7.2 in 25 mmol/L ammonium bicarbonate with 3 mmol/L calcium chloride for 5 hours. Following digestion, fresh COLaseIII was added to the solution and digestion continued overnight. Following overnight digestion, tissues were homogenized at a pulse of 20/20, 40%, at 10 pulses. Peptides were dried and desalted using a C18 StageTip, followed by a C18 ZipTip (EMD Millipore) as per manufacturer’s protocol.

Peptides were separated and analyzed on an EASY nLC 1200 System (Thermo Fisher Scientific) in-line with the Orbitrap Exploris 480 (Thermo Fisher Scientific) with instrument control software v. 4.2.28.14. Two micrograms of peptides were pressure loaded onto a C18 reversed-phase column (Acclaim PepMap RSLC, 75 µm × 25 cm (2 µm, 100 Å) Thermo Fisher Scientific cat. # 164941) and separated using a gradient of 0% to 35% B in 180 minutes (solvent A: 5% ACN and 0.2% formic acid; solvent B: 80% ACN and 0.2% formic acid) at a flow rate of 300 nL/minutes. Mass spectra were acquired in data-dependent mode with a high-resolution (60,000) Fourier transform mass spectrometry survey scan, mass range of *m*/*z* 375 to 1,700, followed by MS/MS of the most intense precursors with a cycle time of 3 seconds. The automatic gain control was set to 300% for the survey MS scan and 100% for the MS/MS scan. HCD fragmentation was performed with a precursor isolation window of 2 *m*/*z*, a maximum injection time of 120 ms, and HCD collision energy of 33%. The MS/MS scan was acquired at 15,000 resolution. Monoisotopic precursor selection was set to “peptide.” Precursors within 10 ppm mass tolerance were dynamically excluded from resequencing for 20 seconds. Advanced peak determination was enabled. Precursor ions with charge states that were undetermined, 1, or >7 were excluded. A target list was included in the method, if no target mass was found, a dependent scan was performed on the most intense ion.

### Data analysis following LC-MS/MS

For protein identification and quantification, the LC-MS/MS data were searched using the MaxQuant platform and normalized with the label-free quantitation (LFQ) algorithm (34–36). Data were searched against a custom ECM database and a database of common contaminants. The FDR, determined using a reversed database strategy, was set at 1% at the protein and peptide level. The “LFQ” feature was used with “match between runs” enabled for those features that had spectra in at least one of the runs. The “stabilize large ratios” feature was enabled, and “fast LFQ” was disabled. A 4.5 ppm mass tolerance was used. A minimum ratio count of 2 was required for quantification with at least one unique peptide. Parameters included variable protein N-terminal acetylation, oxidation of methionine, and hydroxyproline modifications.

The protein groups text files from the MaxQuant search results were processed in Perseus v1.6.14.0 (RRID: SCR_015753; ref. 37). Protein groups were filtered to remove those only identified by a modified peptide and those matching the reversed database. The raw protein intensities were log_2_ transformed. This yielded 37 proteins. Data in the hydroxyproline and modification specific peptides tables were filtered and log_2_ transformed in the same manner as the protein group table, with 123 unmodified and 289 hydroxyproline peptides identified. These data are provided in Supplementary Table S3.

### Statistical analysis

Imaging data were quantified using SCiLS Lab Version 2023 (Bruker Corporation), and peak intensities were normalized to total ion count. Categorical variables were reported as proportions, and continuous variables were reported as means (+SD) unless otherwise specified. Comparisons of measurements between groups were performed using nonparametric Mann–Whitney tests and ROC curve generation. Statistical analyses were performed using Prism 9.1.0 (GraphPad Software, RRID: SCR_002798). Significant differences were indicated by *P* < 0.05. No corrections for multiple testing were implemented. Normal adjacent tissue cores were omitted from any statistical analysis. Glycans were divided into categories with respect of their structural characteristics (Supplementary Fig. S1). For determining the contribution of each *m*/*z* value to the variations between MET and NED sample cohorts, peak intensity values were used in principal component analysis (PCA) plots, partial least squares discriminant analysis (PLS-DA) plots, and variable importance in projection scores.

For clinical data, patients were stratified by NED and MET status, and baseline demographics and clinical characteristics, including medians, IQRs, and proportions, were tabulated and compared using Wilcoxon, *χ*^2^, or Fisher exact tests as appropriate using the gtsummary R package. Stage and grade were included within a logistic regression model of metastasis status as linear covariates. The AUROC for this logistic regression was estimated using 10 repeats of 10-fold cross validation, and the mean of the out of sample AUC and SD were reported. The AUC for predicting metastasis using stage and grade is 0.719, with SD 0.063. Additional clinical characteristics for tumor stage and grade, OR, and confidence interval for stage and grade, and sites of metastasis are provided in Supplementary Tables S4–S6.

### Data availability

The raw data supporting the conclusion of this article will be made available by the authors upon request, without undue reservation.

## Results

### N-glycan MALDI-MSI analysis of NED and MET TMAs

**Figure 1:**
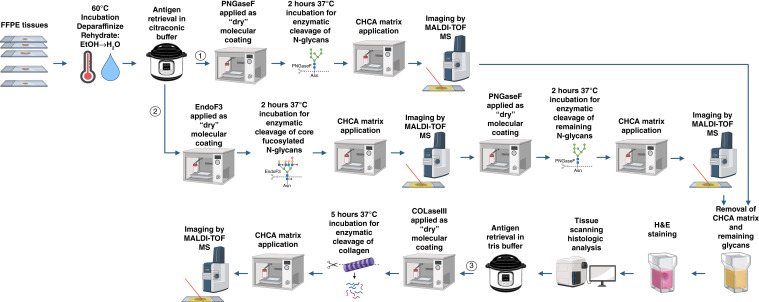
Workflow protocols for sample preparation. For N-glycan analysis, FFPE slides were deparaffinized and rehydrated before undergoing antigen retrieval in citraconic buffer. **PNGaseF (1)** was applied as a “dry” molecular coating before a 2-hour incubation at 37°C. CHCA matrix was applied, and imaging of N-glycans was performed. For analysis of core- vs outer-arm fucosylated N-glycans, enzyme **EndoF3 (2)** was dialyzed before application, and imaging of EndoF3-digested N-glycans was performed before subsequent PNGaseF digestion and imaging of remaining N-glycans. Following glycan imaging, CHCA matrix was removed from tissues, and samples were histologically analyzed following H&E staining. Tissues underwent a second antigen retrieval with tris buffer before **COLaseIII (3)** was applied as a “dry” molecular coating. Slides were incubated for 5 hours at 37°C. CHCA matrix was applied, and imaging of collagen/ECM peptides was performed.

A unique clinical outcome TMA cohort of patients with prostate cancer was constructed from initial prostatectomy pathology blocks for two sets of subjects after 5 to 10 years of follow up: those that had no evidence of disease (NED; *n* = 73) and those that eventually had a documented prostate cancer metastasis (MET; *n* = 51). Clinical properties of the cohort are summarized in Table 1, and representative H&E stains of each TMA are provided in Supplementary Fig. S1. The 1-cm prostate cancer tumor core sizes are larger than most TMA constructs to better capture the tumor and stroma heterogeneity characteristic of prostate cancer tissues. Therefore, most cores have some stromal components. The five TMA slides were sequentially evaluated by N-glycan and collagen/ECM MALDI-MSI analysis using established protocols (27, 29, 38), as summarized in Fig. 1. For N-glycan MALDI-MSI, two strategies were used, one using PNGaseF only to profile released N-glycans, and the other used sequential EndoF3 and PNGaseF digestions.

**Figure 2:**
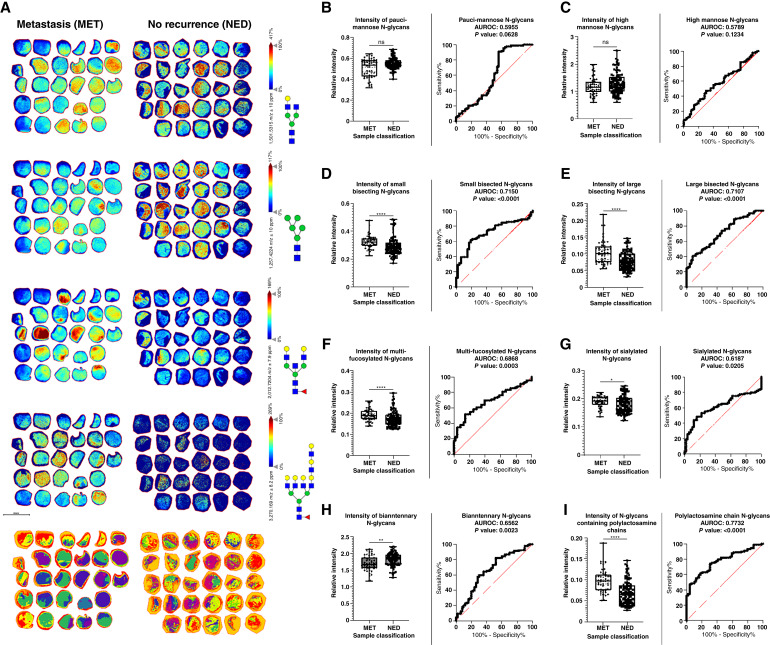
Glycan imaging data from TMA comparing metastasis vs. no recurrence. **A,** MALDI-MSI images of TMAs from metastasis (MET) category and no recurrence (NED) category. N-glycan images representative of differentiating expression of select N-glycan categories, followed by segmentation analysis of 152 detected N-glycans. **B–I,** Box plot and ROC curves for each category of N-glycan detected within each group of TMAs. *t* test results shown on each boxplot.

For PNGaseF only, MALDI-MSI of the five TMA slides resulted in the cumulative detection of 152 N-glycan *m*/*z* values, with representative images shown in Fig. 2A and Supplementary Fig. S2, and structural compositions for each in Supplementary Table S1. Using the individual peak intensity values for each N-glycan species, two analysis strategies were used. The first one involves grouping the N-glycans based on their structural compositions, listed in Supplementary Table S1, and defined in Supplementary Fig. S3. As shown in Fig. 2B–I, these groups include known prostate cancer tumor N-glycan classes like pauci-mannose, oligo-mannose, and branched multi-fucosylated, and more stromal N-glycan classes like small bisecting N-acetylglucosamine (bisecting GlcNAc) and biantennary (22, 24, 39). Other structural classes like sialylated N-glycans, large bisecting GlcNAc, and N-glycans containing polylactosamine chains may be present in tumor and stroma. Most detected glycans were included in multiple groupings depending on their structures. Peak intensities were used to compare NED and MET conditions (Fig. 2B–I), and accompanying AUROC curve analysis was also generated. For NED tissues, most notable was the higher levels of biantennary N-glycans, and slightly elevated levels of pauci- and oligo-mannose structures (Fig. 2B, C, H) with AUROC ranges between 0.5789 and 0.6562. Most structural classes indicated higher levels of N-glycans in the MET cohort, typified by the presence of larger branched multiantennary and bisecting GlcNAc structures, as well as fucosylation, sialylation, and polylactosamine content (Fig. 2D–G, I) with AUROC values ranging between 0.6187 and 0.7732. Cumulative tumor stage and Gleason scores for both cohorts were assessed for their ability to discriminate NED from MET tissues, yielding an AUROC value of 0.719 (Supplementary Table S4). Given that current tissue-based genetic assays perform in the 0.7 to 0.8 range (9), the goal for identifying potential discriminating N-glycan markers should have AUC values at or above this range.

**Figure 3:**
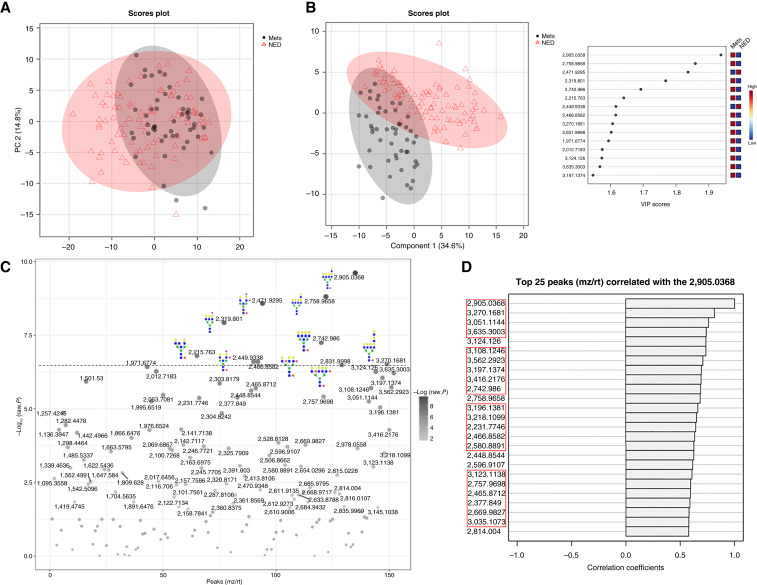
Statistical analysis of detected N-glycans in each category of TMA. **A,** PCA score plot of all 152 N-glycans. **B,** PLS-DA plot and corresponding importance feature plot. **C,***t* test plot of NED vs. MET with structures shown for the top 10 most significant peaks. **D,** Pattern search graph showing the top 25 peaks correlated with 2905.0368 (Hex8dHex1HexNAc7), *m*/*z* values of N-glycans with at least one or more fucose shown in red boxes. VIP, variable importance in projection.

The ability of individual N-glycan species to distinguish NED and MET cohorts using relative peak intensities was evaluated initially by PCA (Fig. 3A), PLS-DA (Fig. 3B), and by AUROC (Supplementary Fig. S4). Fifteen N-glycans had AUROC values between 0.7 and 0.8 (Supplementary Fig. S4). A *t* test plot shown in Fig. 3C highlights the top 10 N-glycan structures that were most significant in distinguishing NED and MET cohorts, primarily comprised of glycans with multiple branches and/or bisecting GlcNAc structures, sialic acid, and fucose modifications. The most differential N-glycan was at *m*/*z* = 2,905 *m*/*z* (Hex8dHex1HexNAc7), a tetra-antennary glycan with one polylactosamine chain and a core fucose structure, with an AUROC of 0.804. For most prostate cancer tissues, this glycan localizes to primarily stroma regions. Pattern hunter analysis (MetaboAnalyst) shown in Fig. 3D revealed that 20 of the top 25 peaks correlating with the levels of the 2,905 *m*/*z* glycan contained at least 1 fucose within their structure, prompting further investigation into the role of fucosylation within each cohort.

### Sequential glycosidase digest and MALDI-MSI to identify core-fucosylated N-glycans

**Figure 4:**
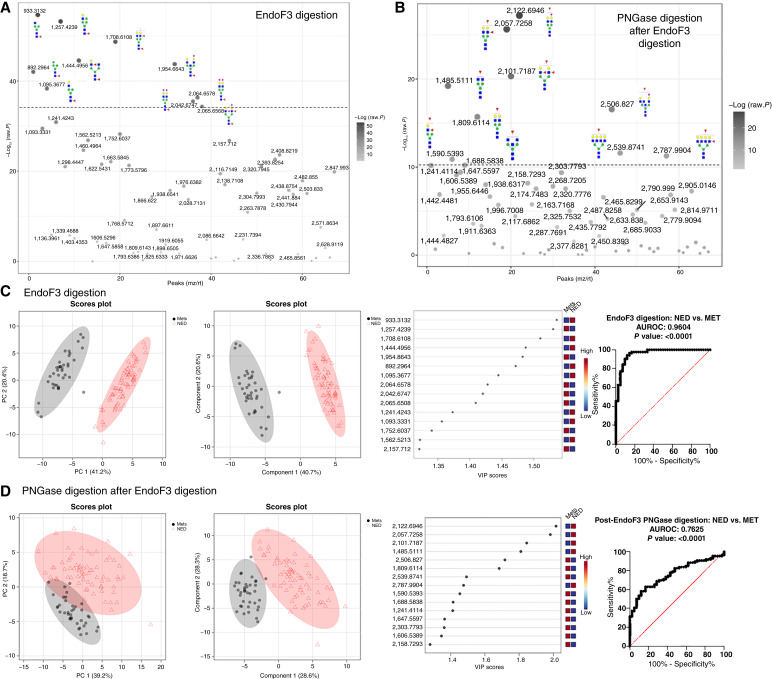
Statistical analysis of detected N-glycans in each category of TMA following EndoF3 and PNGaseF digestion. **A,***t* test plot of detected N-glycans following EndoF3 digestion. Structures shown for top 10 most significant N-glycans (**B**) *t* test plot of detected N-glycans following PNGaseF digestion. Structures shown for top 10 most significant N-glycans **C,** Left-Right: PCA, PLS-DA plots for detected N-glycans following EndoF3 digestion. ROC curve showing NED vs. MET after EndoF3 digestion **D,** Left-Right: PCA, PLS-DA plots for detected N-glycans following PNGaseF digestion. ROC curve showing NED vs. Mets after PNGaseF digestion.

Adjacent sections of each TMA were digested with EndoF3 to specifically release N-glycans with a core fucose attached to the GlcNAc bound to asparagine on the carrier glycoproteins. The GlcNAc and fucose remain attached to the protein, resulting in a glycan product that is 349 mass units less than the parent structure when analyzed by MALDI-MSI. Next, the slides were washed and sprayed with PNGase F to release the remaining N-glycans, followed by another MALDI-MSI run. This approach specifically can distinguish core-fucosylated glycans from those that have fucose attached to antennae. Digestion with EndoF3 and subsequent MALDI-MSI resulted in the detection of 76 core-fucosylated N-glycans, with product masses listed in Supplementary Table S1. Statistical analysis via *t* test found that structures with significantly different expression within each cohort were mostly biantennary, with only 1 of the top 10 structures containing a bisecting GlcNAc (Fig. 4A). A *t* test performed after digestion with PNGase F following EndoF3 digestion revealed more multiantennary structures and structures containing a bisecting GlcNAc within the top 10 most significantly different peaks (Fig. 4B). Representative images corresponding to *m*/*z* values associated with core-fucosylated N-glycans show a higher intensity in the MET cohort when compared with the NED cohort (Supplementary Figs. S5 and S6). The N-glycan with the most significantly different expression in MET versus NED tissues after EndoF3 digestion is the structure corresponding to *m*/*z* + 933, the parent structure being a small biantennary core-fucosylated N-glycan (Hex3dHex1HexNAc3). After subsequent PNGase digestion, the N-glycan with the most significantly different expression in MET versus NED tissues is the structure corresponding to *m*/*z* = 2,122 (Hex5dHex1HexNAc4NeuAc1) Analysis via PCA (Fig. 4C), PLS-DA (Fig. 4D), and AUROC indicated that EndoF3 digestion yielded more significant differentiation of N-glycan profiles between MET tissues and NED tissues than PNGaseF alone. AUROC analysis of the total EndoF3 glycan peak list (Fig. 4C) and individual core-fucosylated glycans (Supplementary Fig. S7) yield values of 0.95 and greater. Overall, the prostate cancer tissues in the MET cohort had more N-glycan structures with core-fucose modifications than in NED tissues.

### Predictive N-glycan biomarkers extend beyond N-glycans found within prostate cancer tumors

N-glycan MALDI-MSI analysis was also done on whole tissues from which the cores of the TMA cohorts were made. Whole tissues were digested by PNGase with the purpose of investigating the distribution of distinguishing N-glycan structures with more histologic variety than what is seen in the tissue cores. These whole tissues exhibited a variety of prostate cancer characteristics, including regions of different types of tumors and various ductal regions. The site in each tissue from which the TMA core was taken is easily identified, and other histopathologic features are further annotated on each tissue (Supplementary Fig. S8A and S8B). The N-glycan structures shown in Supplementary Fig. S8C are consistent with those detected in MET and NED TMA cohorts. Segmentation analysis of each whole tissue further demonstrated distinct N-glycan profiles in MET tissues when compared with NED tissues (Supplementary Fig. S9). Oligo-mannose N-glycans were localized primarily to the tumor region of the larger tissues, consistent with previous findings that oligo-mannose N-glycans are elevated in prostate tumors (40). Additionally, biantennary N-glycan structures were found to be more stroma localized throughout the NED full tissue, consistent with findings within the TMA cohort. Finally, larger, more complex N-glycan structures were shown to have overall higher expression within the MET tissues compared with the NED tissues, specifically within tumoral regions and around the TMA sampling site, consistent with previous findings with regard to an increase in the expression of large, heavily modified structures within the MET cohort. These findings confirm that the TMA glycan data can be representative of the N-glycan profiles detected in the larger tissue slides.

### Stroma and ECM targeted proteomic analysis by MALDI-MSI after COLaseIII digestions

**Figure 5:**
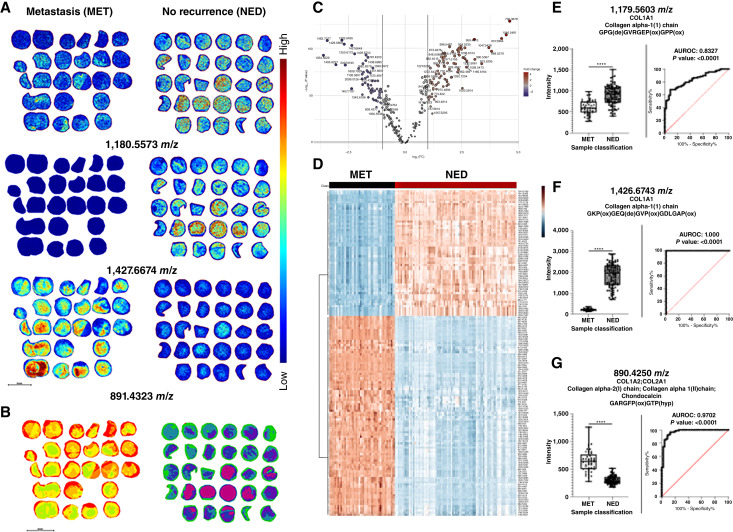
Collagen/ECM imaging data from TMAs comparing metastasis vs. no recurrence. **A,** Representative MALDI-MSI images of TMAs from metastasis (MET) category and no recurrence (NED) category. **B,** Segmentation analysis of 294 detected peptides. **C,** Volcano plot created from list of 294 detected peptides **D,** Heatmap of top 150 significantly different detected peptides. **E,** Box plot and ROC curve for 1179.5603 *m*/*z*. **F,** Box plot and ROC curve for 1426.6743 *m*/*z*. **G,** Box plot and ROC curve for 890.4250 *m*/*z*.

A previous study using nine paired prostate cancer tissues with multiple tumor grade and benign pathologies digested with a bacterial collagenase protease had reported detection of a total of 19 different collagen types and other ECM proteins following using combined MALDI-MSI and tandem LC-MS approaches (27). Different collagen types could distinguish tumor from benign regions, and in particular, specific proline hydroxylation modifications on the collagens influenced localization (37). Therefore, each of the TMA slides evaluated for N-glycans was further analyzed using COLaseIII, followed by MALDI-MSI analysis. As shown in Fig. 5 and Supplementary Fig. S10, there were dramatic differences in ECM peptide composition between the MET and NED samples, as shown for *m*/*z* values 1179.5603, 1426.6743, and 890.425. A list of 294 MALDI-MSI detected peptides was created by manual peak identification (Supplementary Table S2), and three representative MALDI-MSI image profiles are shown in Fig. 5A, and Supplementary Fig. S10. Statistical analysis revealed many peptides with potential to distinguish MET and NED tumor tissues, as shown by segmentation analysis (Fig. 5B), volcano plot (Fig. 5C), a *t* test graph, and PCA/PLS-DA analyses (Supplementary Fig. S11). Furthermore, a heatmap depicting the top 150 most significant peaks revealed a stark difference in peptide composition between the MET and NED cohorts (Fig. 5D). The same three peptides shown in Fig. 5A were used for ROC analysis, as shown in Fig. 5E, with near total separation for two peptides (ROC = 1.0, *m*/*z* 1426.6743 and ROC 0.97, *m*/*z* = 890.425). These annotated amino sequence spectra of these three peptides and one other are provided in Supplementary Figs S12–S15 and linked directly with their corresponding *m*/*z* values in the MALDI-MSI data. In general, peptides with lower *m*/*z* values were found to be more upregulated in MET tumors, whereas peptides with larger *m*/*z* values were found to be more upregulated in NED tumors.

**Figure 6:**
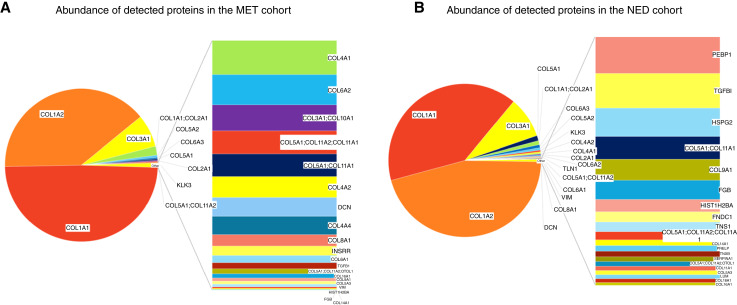
Analysis of collagen/ECM profile of NED vs. MET cohort from data obtained by LC-MS/MS. **A,** Pie chart representations of abundance (peak intensity and number of peptides identified via LC-MS/MS) of all detected protein species within the MET cohort. **B,** Pie chart representations of abundance of all detected protein species within the NED cohort.

To determine the corresponding peptide sequences of the MALDI-MSI, proteomic analysis was done on homogenized tissue cores from NED and MET slides using LC MS/MS. Proteomic LC MS/MS analysis of collagenase-digested peptides from each cohort revealed a list of specific protein species that have been detected in NED or MET tissues. Out of all detected proteins, five proteins were found only in the MET tumor cohort (Fig. 6A), whereas 12 proteins were found exclusively in the NED cohort (Fig. 6B). In both cohorts, 27 protein species were detected to some degree (Fig. 6; Supplementary Fig. S16A). When quantifying types and abundances of posttranslational modifications in all detected peptide sequences, the quantity of modifications between MET tissues and NED tissues had no significant differences in abundances. Quantification of the number of peptides corresponding to each detected protein within both cohorts revealed that the most abundant protein species in MET tissues was collagen type 1 alpha (I) chain (COL1A1), followed by collagen type 1 alpha (II) chain (COL1A2) and collagen type 3 alpha (I) chain (COL3A1) as second and third most abundant, respectively. In NED tumor tissues, which had far more variability in protein species detection, the top three most abundant species were also COL1A2, COL1A1, and COL3A1, respectively. There is also more variability in the type and number of proteins in each cohort (Fig. 6). Digestion of TMAs with COLase III resulted in the detection of 294 peptides (Supplementary Table S3).

**Figure 7:**
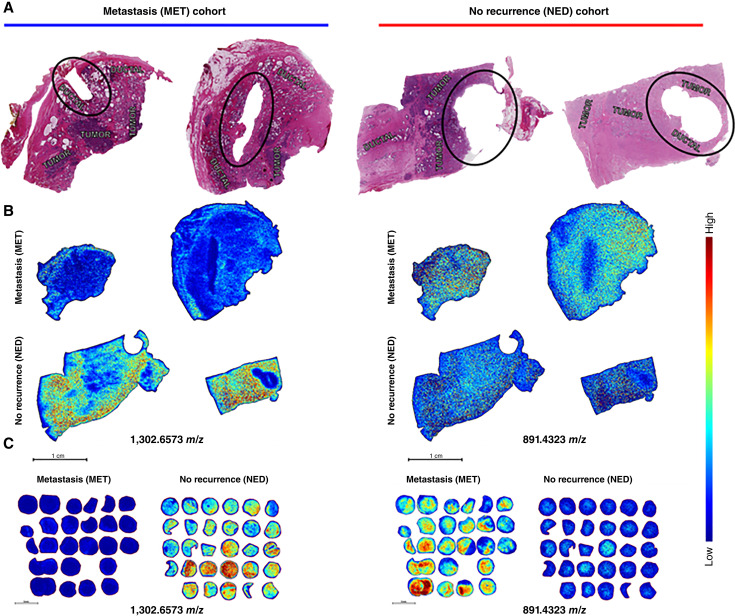
Collagen/ECM MALDI-MSI data from full tissue samples (**A**) H&E-stained images of tissue from metastasis (MET) category and no recurrence (NED) category. Region where TMA was sampled is circled in black. Note that the MET tissue is the same H&E image used in Supplementary Fig. S8A, as N-glycans and collagenase peptides were detected sequentially on the same tissue slide. **B,** Corresponding MALDI-MSI images after digestion with COLaseIII from the two metastasis (MET) tissues (top) and the two no recurrence (NED) tissues (bottom). Corresponding *m*/*z* values for each image are shown to the right. **C,** Representative MALDI-MSI images from the metastasis (MET) TMA and no recurrence (NED) TMA of the same mass corresponding to full tissues. Note that the collagenase peptide image for *m*/*z* = 891.4323 is the same data image used in Fig. 5A for this ion.

Analysis of full-size TMA core source tissues with various histologic features revealed similar patterns as the patterns seen in the TMA cohorts (Fig. 7A and B). Although not as distinctly localized within certain histologic features in the same way that N-glycan images of these tissues depict, the peak intensities of detected peptides in images of full tissues correspond to the findings within the imaging results for the TMA cohort (Fig. 7B and C). There are significant underlying changes in collagen types and other ECM proteins in the NED and MET cohorts, highlighted in the segmentation analysis of these full tissues (Supplementary Fig. S17). The collagen/ECM proteins and proline-hydroxylated peptides that are unique to the MET cohort relative to the NED samples are likely to contribute to metastatic prostate cancer progression. Collagen species are a key component of reactive stroma and can act as a substrate for cancer cells while they migrate to different locations. In addition to reactive stroma composition, it has also been theorized that collagen may also play a role in inhibiting cancer cell migration by wrapping around the tumor and restricting its ability to metastasize. Consistent with this knowledge, these results confirm that different peptide species may either potentially promote cancer aggressiveness in the MET tissues or inhibit the further spread of prostate cancer in the NED tissues.

## Discussion

As previously established, aberrant N-glycosylation and changes within the construction of the ECM are known hallmarks for prostate cancer progression (12, 27, 40). These molecular-level changes have previously been poorly understood in the context of predictors for prostate cancer recurrence and metastasis. Here, we document multiple molecular changes within the N-glycome and ECM of two cohorts of prostate cancer TMAs derived from 121 primary prostatectomy samples with clinical outcome follow-up information. A summary table of N-glycan and ECM peptides with AUROC values greater than 0.8 is provided in Supplementary Fig. S18. These results derive from three unique aspects of the experimental design. The most critical component is the composition of the clinical outcome TMA samples. The tissues in the MET cohort came from men with confirmed metastatic prostate cancer, more definitive than a biochemical recurrence designation, and had documented metastasis confirmed by a urologic oncologist. The NED tissues were from men with an average of 10 years of clinical follow-up. Applying to these TMAs the combined MSI workflows that target core fucosylation and the collagen network of the ECM proteome are also unprecedented approaches for prostate cancer. The glycan and ECM protein results highlight the long suspected and evaluated hypothesis that there are molecular signatures in organ confined prostatectomy prostate cancer tissues that indicate the potential of future metastasis. Molecular transformation of the collagen composition, degree of hydroxyproline cross-linking, and glycosylation changes in the ECM would be expected, and this current study represents a blueprint for further study of the cell surface and ECM niche.

It is well-established that an increase in the abundance of core-fucosylated N-glycans has been associated with pathologically advanced prostate cancer (16, 40–42), including evaluation of core-fucosylated PSA in serum and urine (43–45). The results of these studies depicting an increase in the abundance of core-fucosylated structures within the cohort of tissues derived from the MET cohort are consistent with these previous findings. Furthermore, increased detection of core-fucosylated structures within the MET cohort when compared with the NED cohort could be attributed to an overall increase in the abundance of N-glycans within the MET cohort, another indicator associated with prostate cancer progression linked with overall complexity of N-glycan structures (12, 15, 40, 46, 47). There is a clear increase in structural complexity in the MET tissue cohort, which include the addition of multiple fucose, sialic acid, and terminal GlcNAc structures on multi-branched N-glycans. The N-glycan species that were increased in the NED tissues were primarily simpler biantennary structures with and without core fucosylation, which have been previously associated with tissue localizations in the stroma (17, 25, 39).

Changes in the ECM proteome and glycome are requisite to generating a reactive stroma microenvironment, a well-documented change in prostate cancer (18, 19), but remains largely unexplored for biomarkers of aggressive prostate cancer. A small cohort of prostate tissues representing benign conditions and cancers was assessed by collagenase-based MALDI-MSI and LC-MS analysis (27). This study used whole-tissue lysates, yielding deeper levels of peptide detection relative to the current study. The overall results are comparable, as evidenced by detection of the same most abundant collagen types, e.g., COL1A1, COL1A2, and COL3A1, with a total of 14 types detected (COL1–6, COL8–12, COL14, and COL16). Proportionally more COL1A2, COL3A1, and COL5A1 peptides were detected in the NED cohort compared with the MET (Fig. 6), whereas COL1A1 was most abundant in the MET tissues. The previous study (27) highlighted the importance of hydroxyproline content, indicating that the presence of hydroxyprolines on peptides from COL1A1 and COL1A2 determined their histopathologic location in inflammatory regions (less HYP) or tumor (more HYP). As the current cohort was primarily tumor, there was an abundant number of hydroxyproline modified collagen peptides. However, for the most abundant collagen types, the proportion of peptides with hydroxylated prolines compared with all other peptides was roughly the same in both MET (298HYP/852nonHYP, 34.9%) and NED (314HYP/884nonHYP, 35.5%) cohorts. Consistent with the previous study (27), noncollagen proteins that are known to bind and/or interact with collagens were detected, like lumican, vimentin, heparan sulfate proteoglycan, decorin, and TGFβ1. Interestingly, there were four collagenase-digested peptides from PSA/KLK3 detected in the NED and MET tissues, and their location in the PSA crystal structures is on the protein surface. It is feasible that these peptide regions could be responsible for interacting with PSA substrates, like semenogelin-1 and 2, or could be related to PSA acting extracellularly in the tumor microenvironment to proteolytically modify the ECM.

There are some limitations to this discovery cohort. The MET cohort was over-represented with tissues that had higher Gleason scores at time of prostatectomy compared with the NED cohort. Because prostate cancer recurrence can occur many years after prostatectomy, a key to tissue selection was time from prostatectomy. The men in the NED cohort with an average of 15 years of no disease recurrence were key. For most treatment centers, obtaining the follow up data on men after prostatectomy can be challenging, as many men will only have one visit for surgery, and then are seen by clinicians outside that center. All the donor tissues were obtained from a VA hospital; thus, follow-up information was available through the common electronic medical record system used at the VA. This is a clear strength of the cohort, but a weakness for follow-up and further validation of the molecular assays. Although a new validation cohort is being assembled, including an emphasis on incorporating ancestry diversity for donors, obtaining additional samples in any setting still relies on passage of time and availability of clinical records. With the samples and donors already identified, further studies are planned for analysis of preprostatectomy biopsy tissues and expanding the analysis of full-size donor tissues for increased proteomic depth and multiomic analyses.

The glycosylation and collagen peptide imaging of larger size donor tissue samples indicated results consistent with the findings of the TMA imaging studies, especially for collagenase. For N-glycans, there was an increase in the abundance of fucosylated N-glycans outside of the tumor and throughout the stromal regions of larger tissue specimens. Consistent the TMA cohorts, peptides detected in the MET TMA samples were detected only in the MET donor tissues, and vice versa for NED. These changes can be further explored with the intention of uncovering more information about their role in promoting cancer cell migration or protecting surrounding tissue from cancer cell invasion. The use of full size tissues will allow deeper proteome mining with collagenase, as well as new LC-MS glycopeptide analysis workflows that will link the spatial N-glycans to specific protein carriers (48). The glycopeptide approach will use collagenase and trypsin for digestion and incorporate EndoF3 to better distinguish core-fucosylated glycoproteins. The use of other types of proteases is also feasible, as well as targeting of specific sialic acid modifications (35) that can be integrated with the proteomic workflows.

Overall, our results show that alterations in the N-glycome and ECM composition can distinguish prostate cancer tissues from men who developed metastatic disease from those who had no disease recurrence. These molecular-level changes and their effects on prostatic tissue indicate multiple new research directions for determining the risk of prostate cancer progression at the time of prostatectomy, or possibly prior to prostatectomy if adapted to diagnostic biopsy tissues. Furthermore, with the novel exploration of serum and urine using glycomics (39) and proteomics (49), the findings described here could be applied to the use of liquid biopsies for prostate cancer diagnosis and prognosis.

## Supplementary Material

Figure S1 H&E images of TMAs

Figure S2 MALDI Glycan Images with 5 TMAS

Figure S3 Glycan Classification Visual

Figure S4 ROC data for N-glycan biomarkers

Figure S5 MALDI Images from EndoF3 digestion

Figure S6 MALDI Images from EndoF3 digestion with 5 TMAs

Figure S7 ROC Curves for EndoF3 data

Figure S8 H&E and MALDI-MSI images of N-glycans of full tissue samples

Figure S9 Segmentation Analysis of tissues according to N-glycan analysis

Figure S10 Collagenase digested MALDI images with 5 TMAs

Figure S11 PCA graphs from collagenase digestion

Figure S12 Annotated LCMS spectra for COL1A1

Figure S13 Annotated LCMS spectra for COL1A1

Figure S14 Annotated LCMS spectra for COL1A1;COL1A2

Figure S15 Annotated LCMS spectra for COL1A2;COL2A1

Figure S16 Table of all detected ECM peptides following LCMS analysis

Figure S17 Segmentation analysis of data obtained from full tissue COLaseIII digest

Figure S18 Table of biomarker candidates

Table S1 Glycan Peak List

Table S2 Peptide MALDI Peak List

Table S3 Collagenase-digested peptide LCMS list

Table S4 Patient Stage and Gleason Score

Table S5 Patient Statistical Information

Table S6 MET sites

Supplementary Data Figure Legends Figure Legends for Supplementary Data
