# Supplementary figures and images for: Determining the N-Glycan and Collagen/Extracellular Matrix Protein Compositions in a Novel Outcome Cohort of Prostate Cancer Tissue Microarrays Using MALDI-MSI
Source: Cancer Res Commun. 2024 Nov 27;4(11):3036–48. doi: 10.1158/2767-9764.CRC-24-0152 (PMC11600299; doi:10.1158/2767-9764.CRC-24-0152)

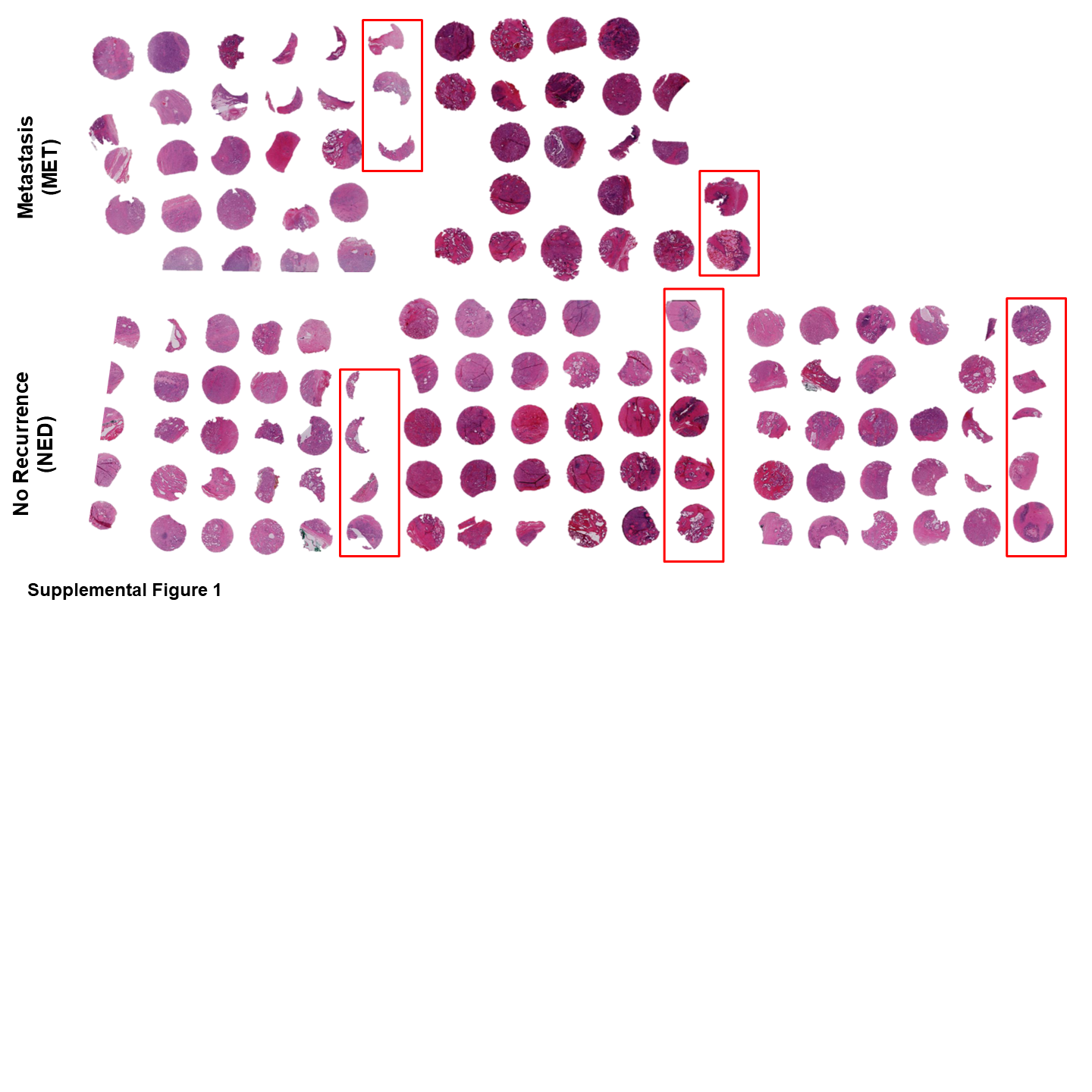

Supplement: Figure S1 — H&E images of TMAs [file crc-24-0152_figure_s1_suppsf1.png]

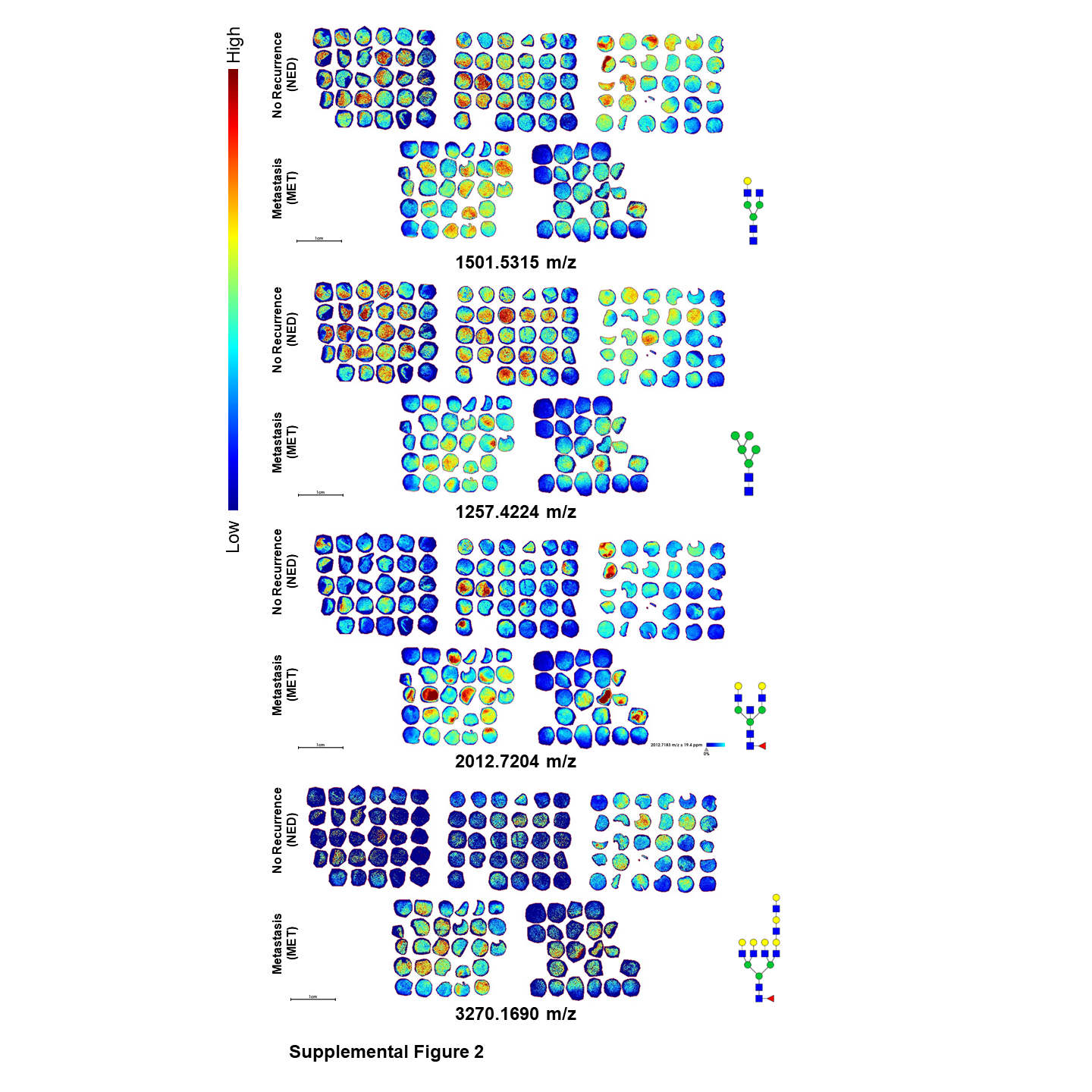

Supplement: Figure S2 — MALDI Glycan Images with 5 TMAS [file crc-24-0152_figure_s2_suppsf2.png]

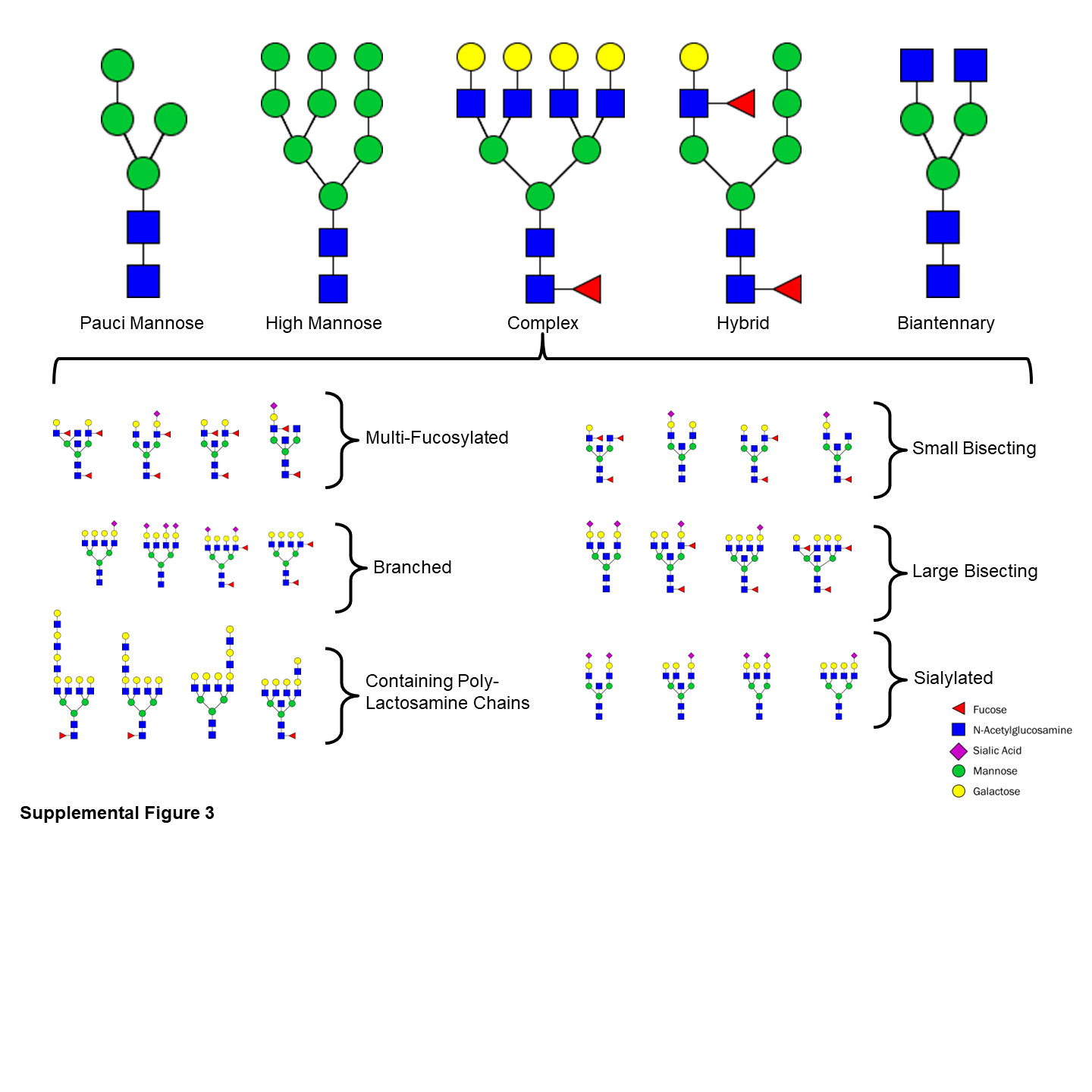

Supplement: Figure S3 — Glycan Classification Visual [file crc-24-0152_figure_s3_suppsf3.png]

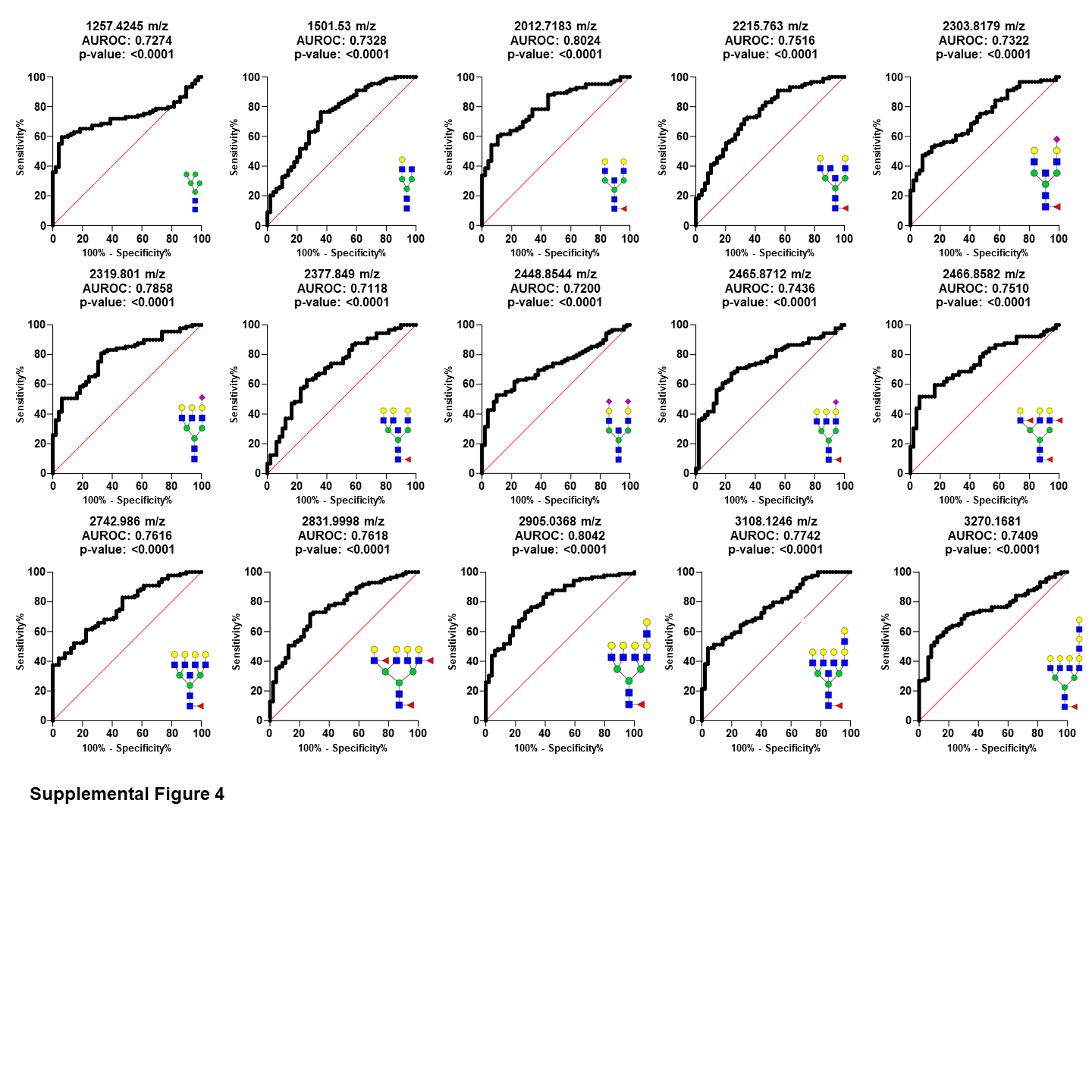

Supplement: Figure S4 — ROC data for N-glycan biomarkers [file crc-24-0152_figure_s4_suppsf4.png]

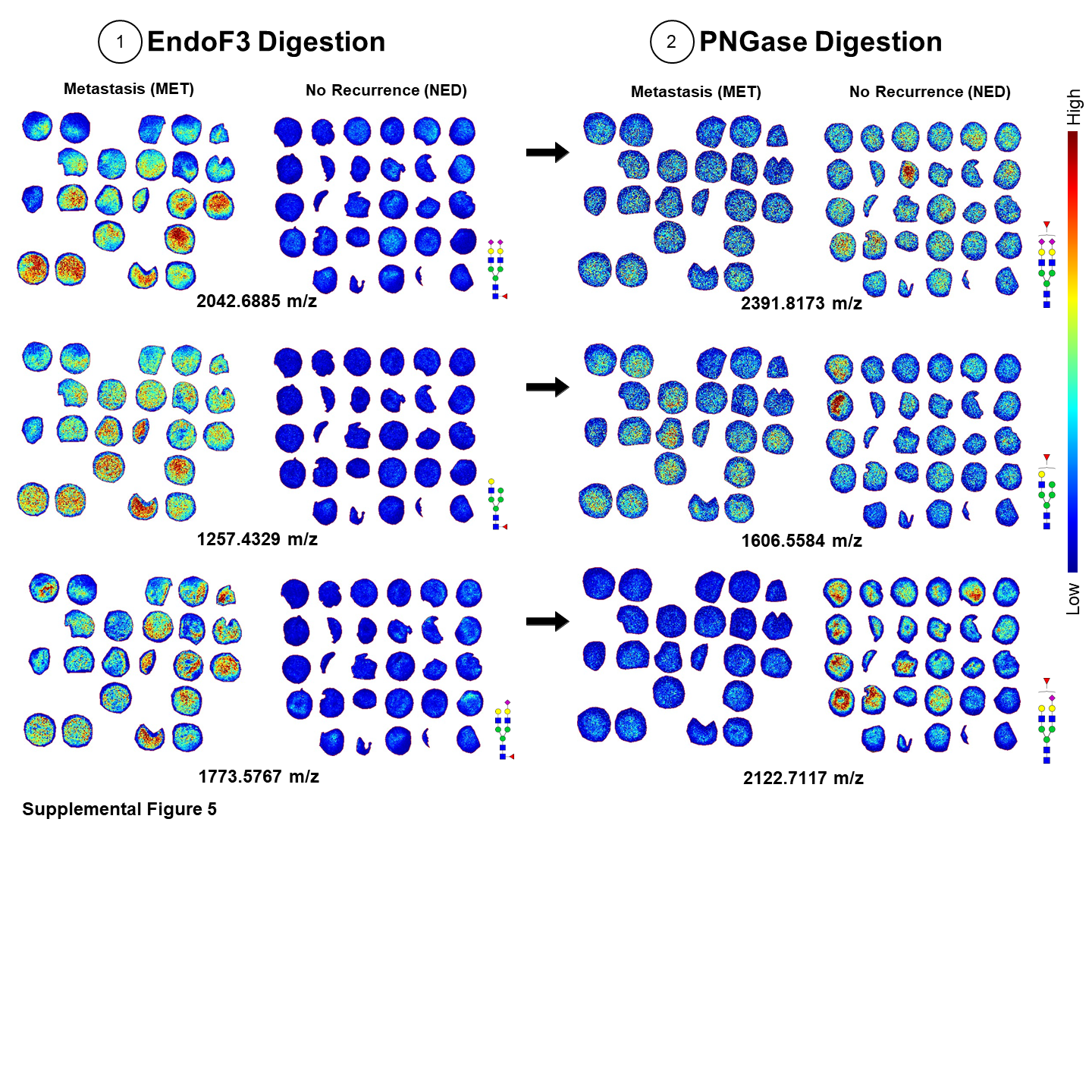

Supplement: Figure S5 — MALDI Images from EndoF3 digestion [file crc-24-0152_figure_s5_suppsf5.png]

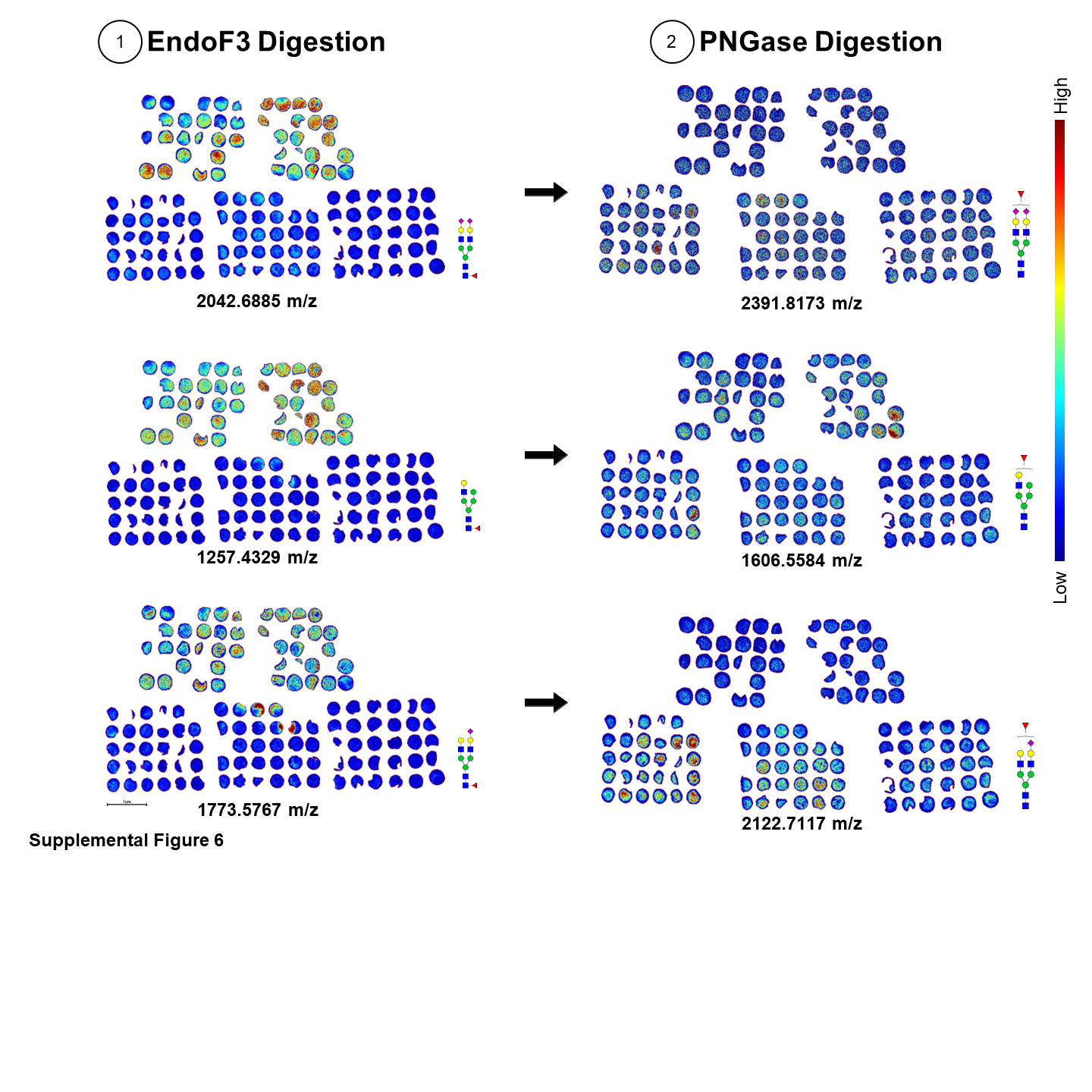

Supplement: Figure S6 — MALDI Images from EndoF3 digestion with 5 TMAs [file crc-24-0152_figure_s6_suppsf6.png]

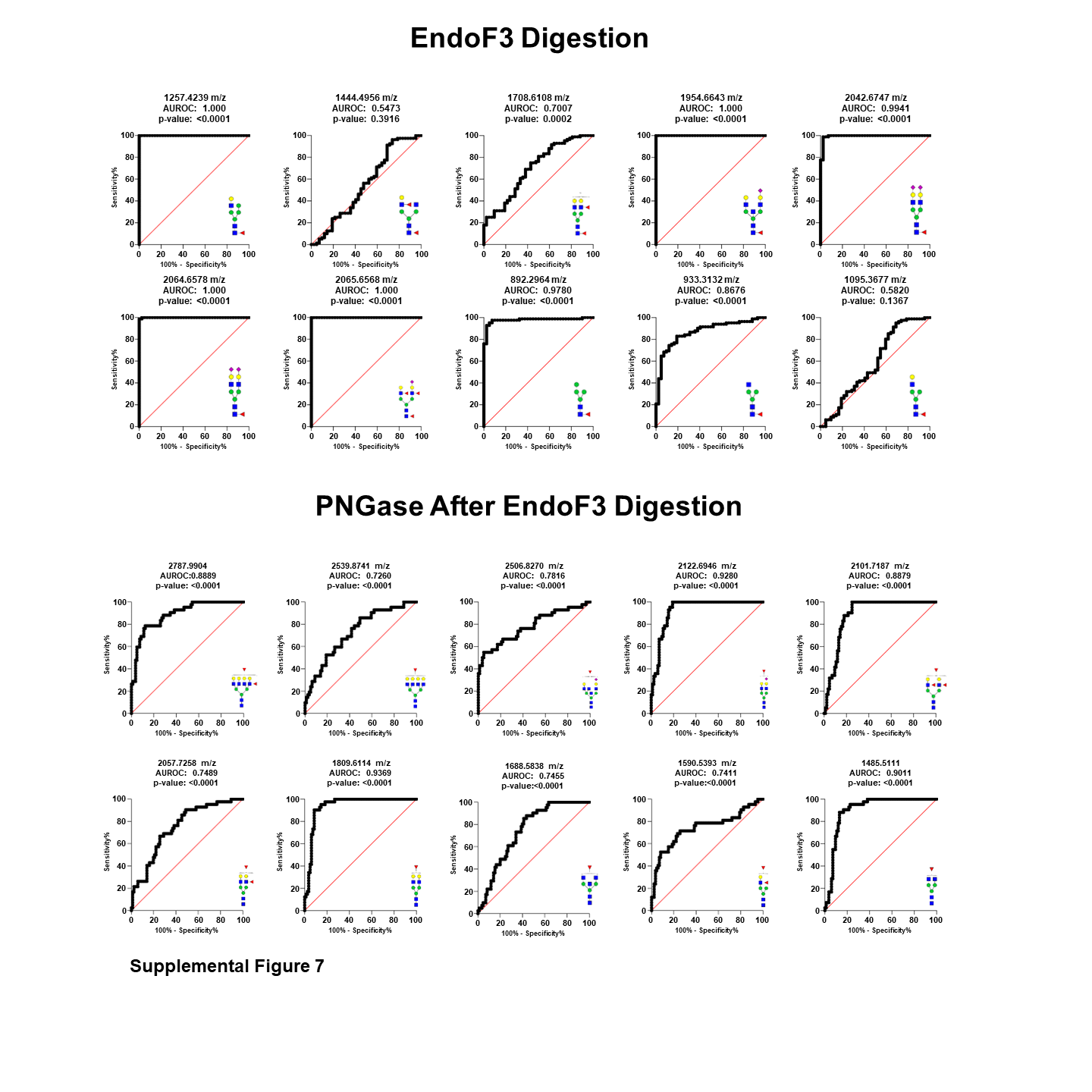

Supplement: Figure S7 — ROC Curves for EndoF3 data [file crc-24-0152_figure_s7_suppsf7.png]

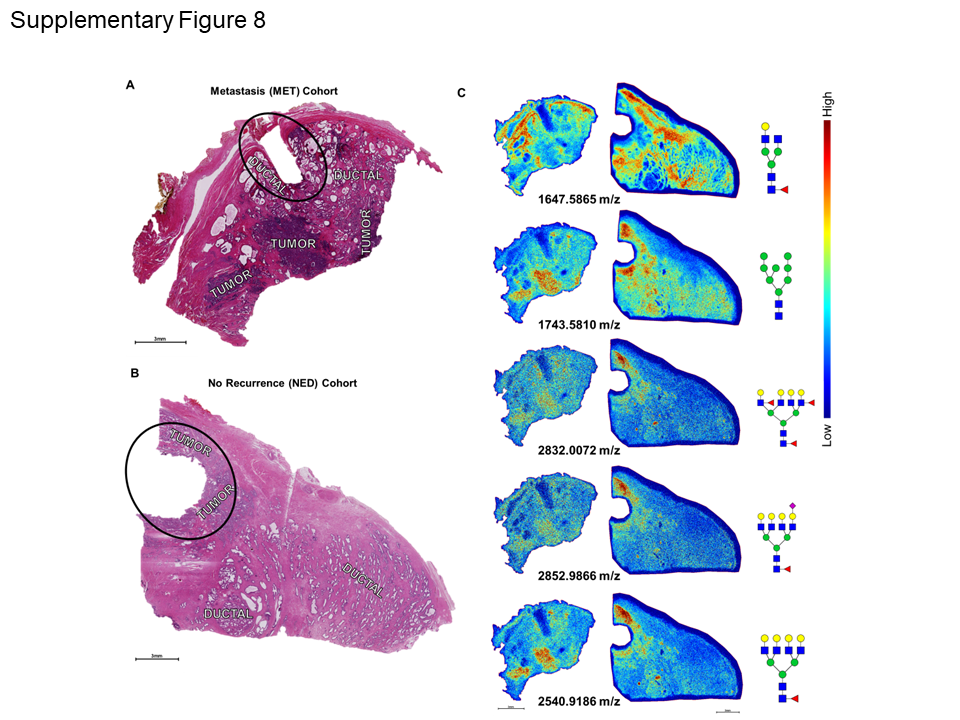

Supplement: Figure S8 — H&E and MALDI-MSI images of N-glycans of full tissue samples [file crc-24-0152_figure_s8_suppsf8.png]

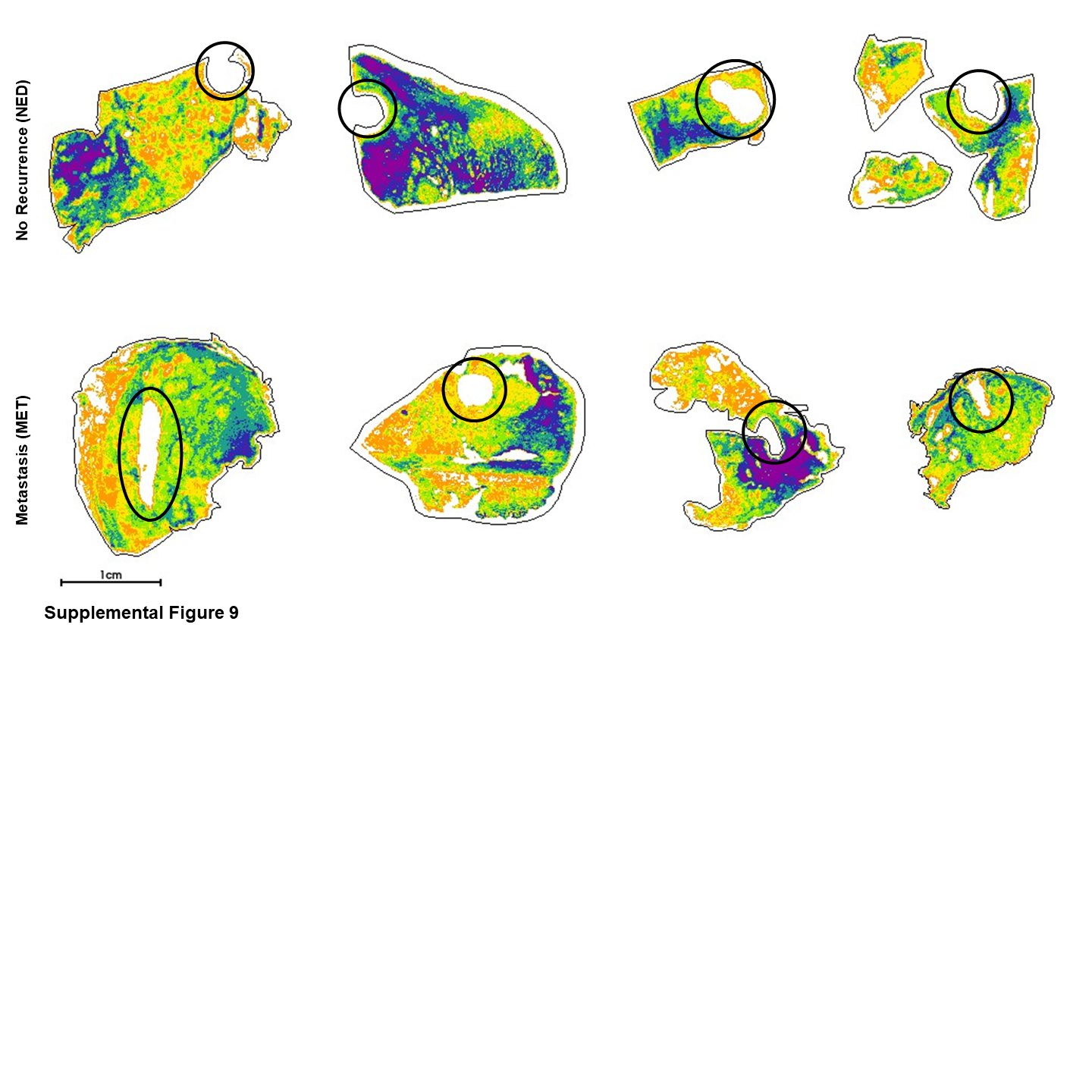

Supplement: Figure S9 — Segmentation Analysis of tissues according to N-glycan analysis [file crc-24-0152_figure_s9_suppsf9.png]

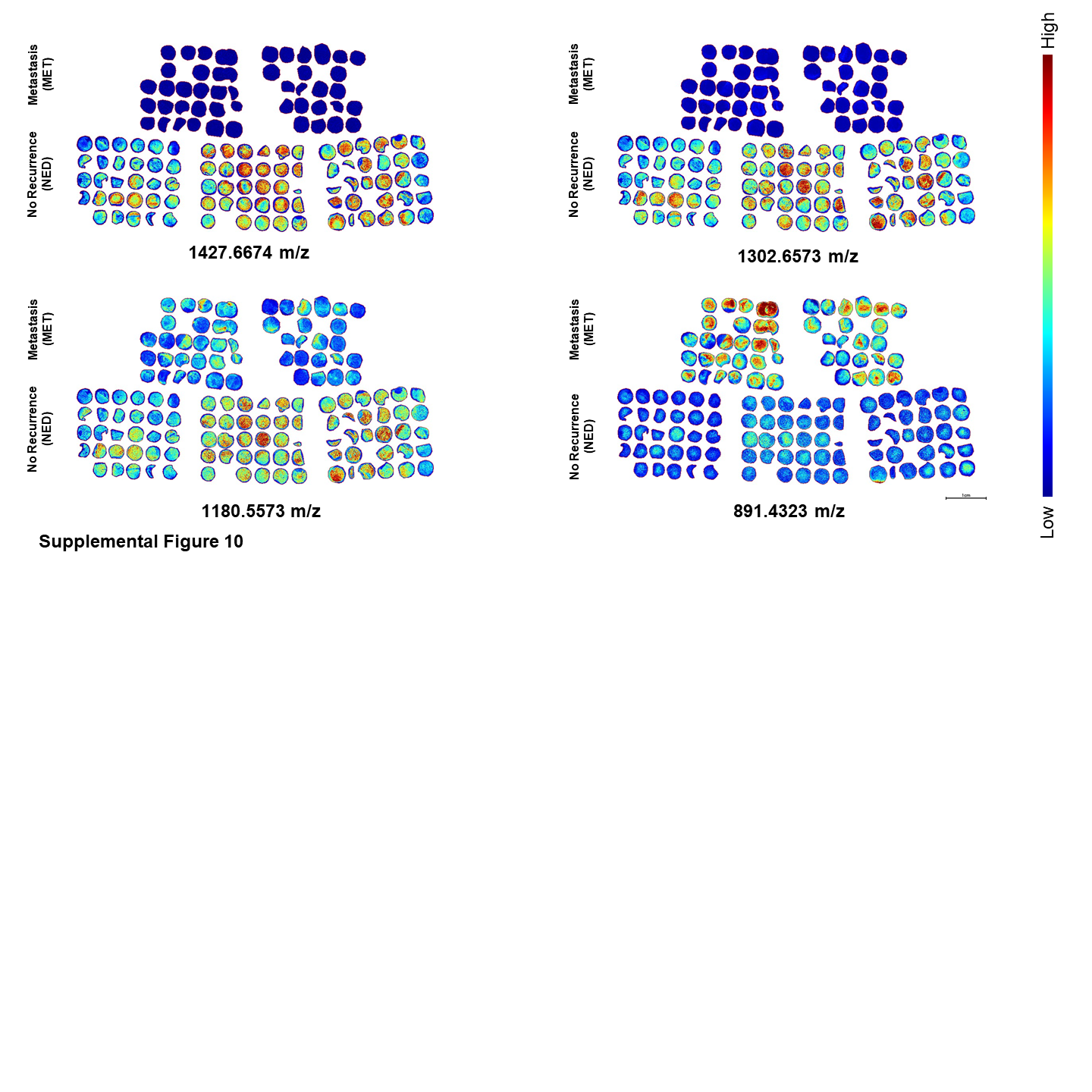

Supplement: Figure S10 — Collagenase digested MALDI images with 5 TMAs [file crc-24-0152_figure_s10_suppsf10.png]

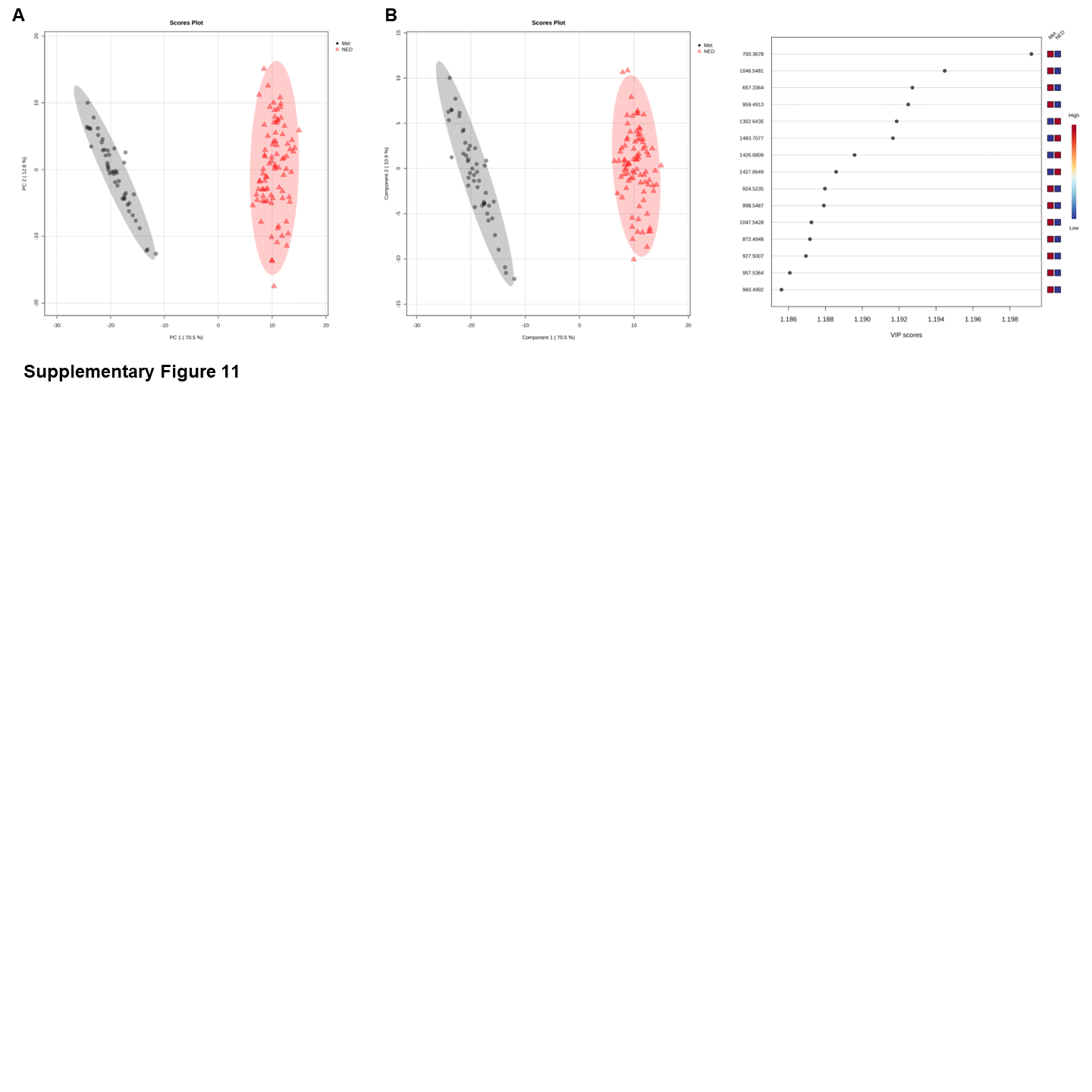

Supplement: Figure S11 — PCA graphs from collagenase digestion [file crc-24-0152_figure_s11_suppsf11.png]

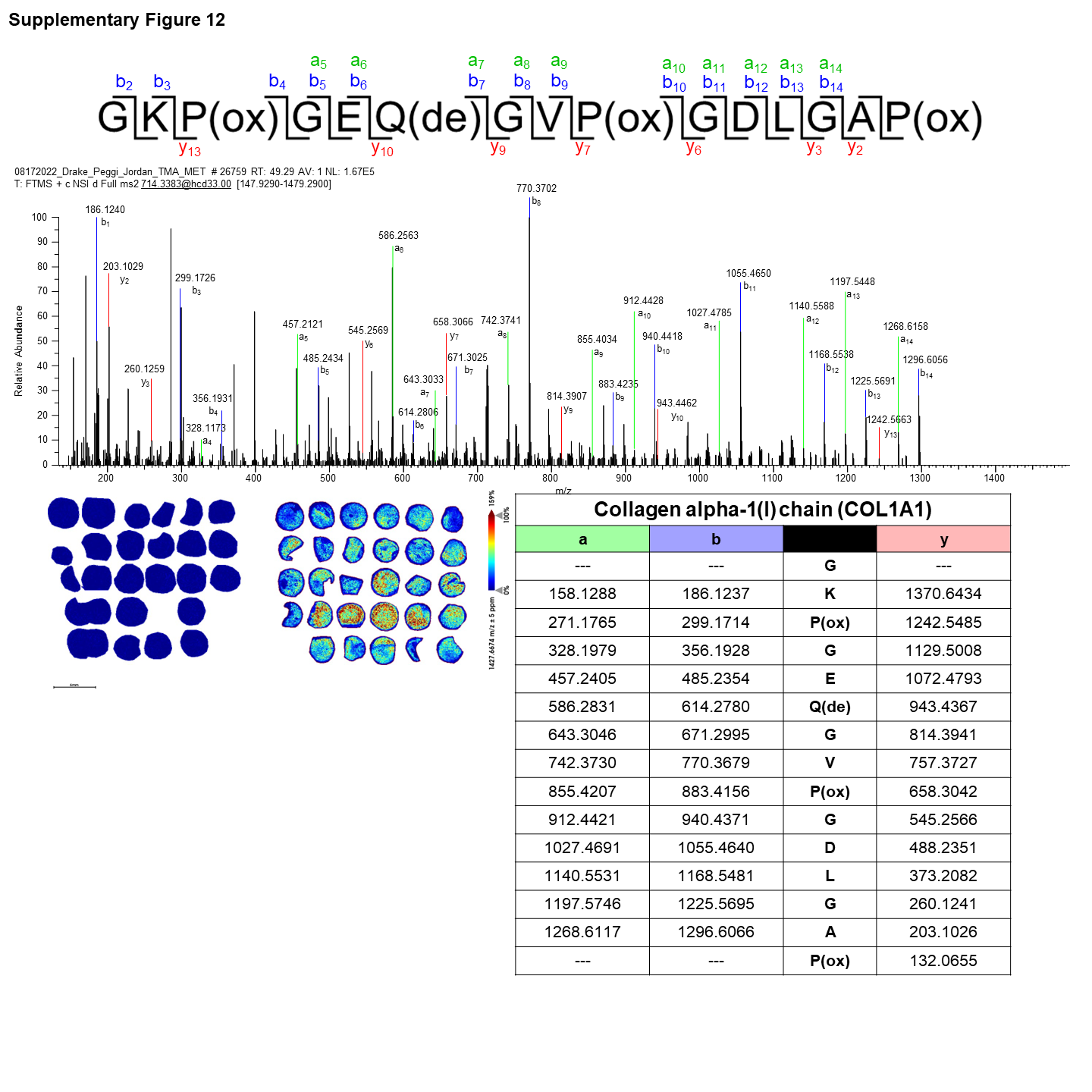

Supplement: Figure S12 — Annotated LCMS spectra for COL1A1 [file crc-24-0152_figure_s12_suppsf12.png]

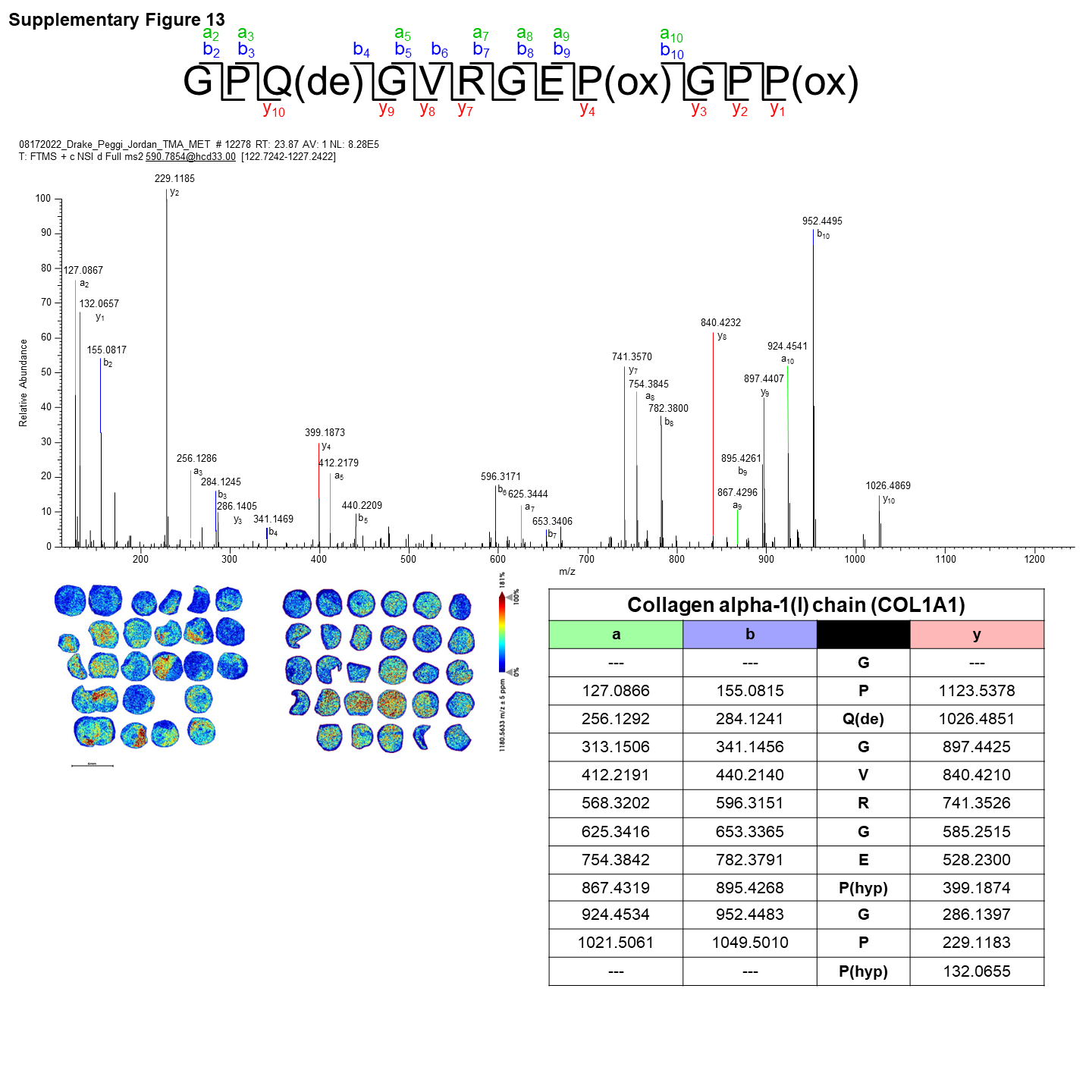

Supplement: Figure S13 — Annotated LCMS spectra for COL1A1 [file crc-24-0152_figure_s13_suppsf13.png]

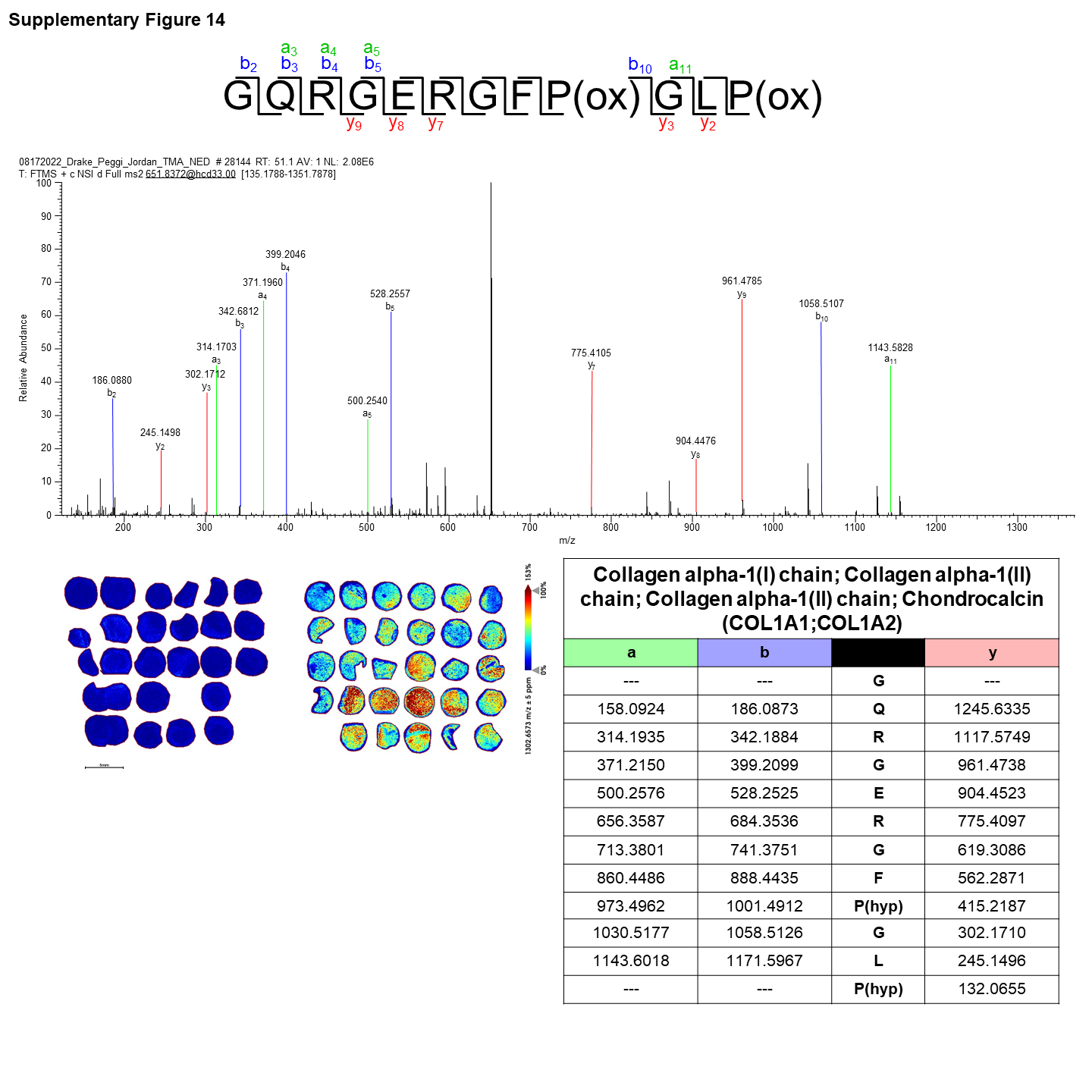

Supplement: Figure S14 — Annotated LCMS spectra for COL1A1;COL1A2 [file crc-24-0152_figure_s14_suppsf14.png]

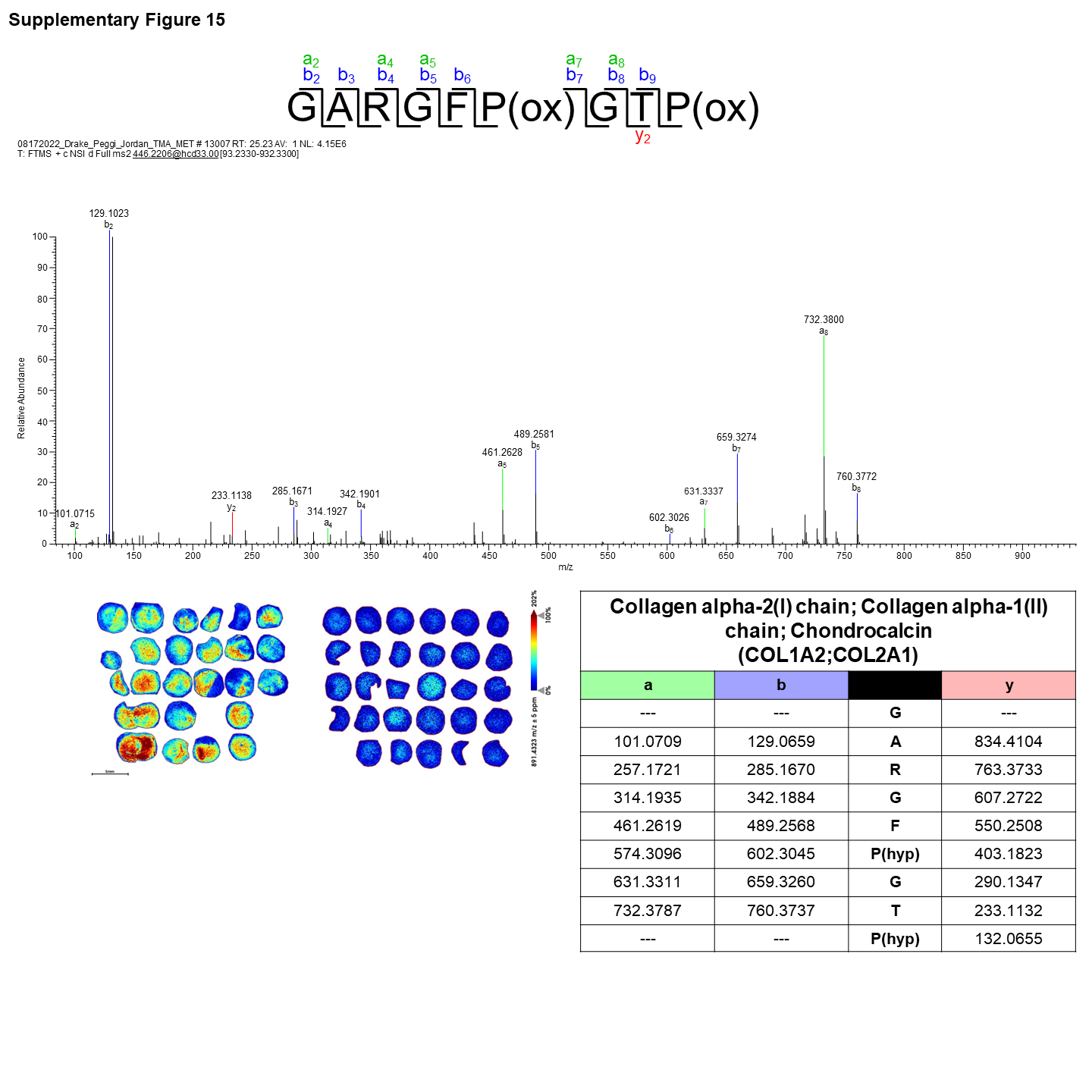

Supplement: Figure S15 — Annotated LCMS spectra for COL1A2;COL2A1 [file crc-24-0152_figure_s15_suppsf15.png]

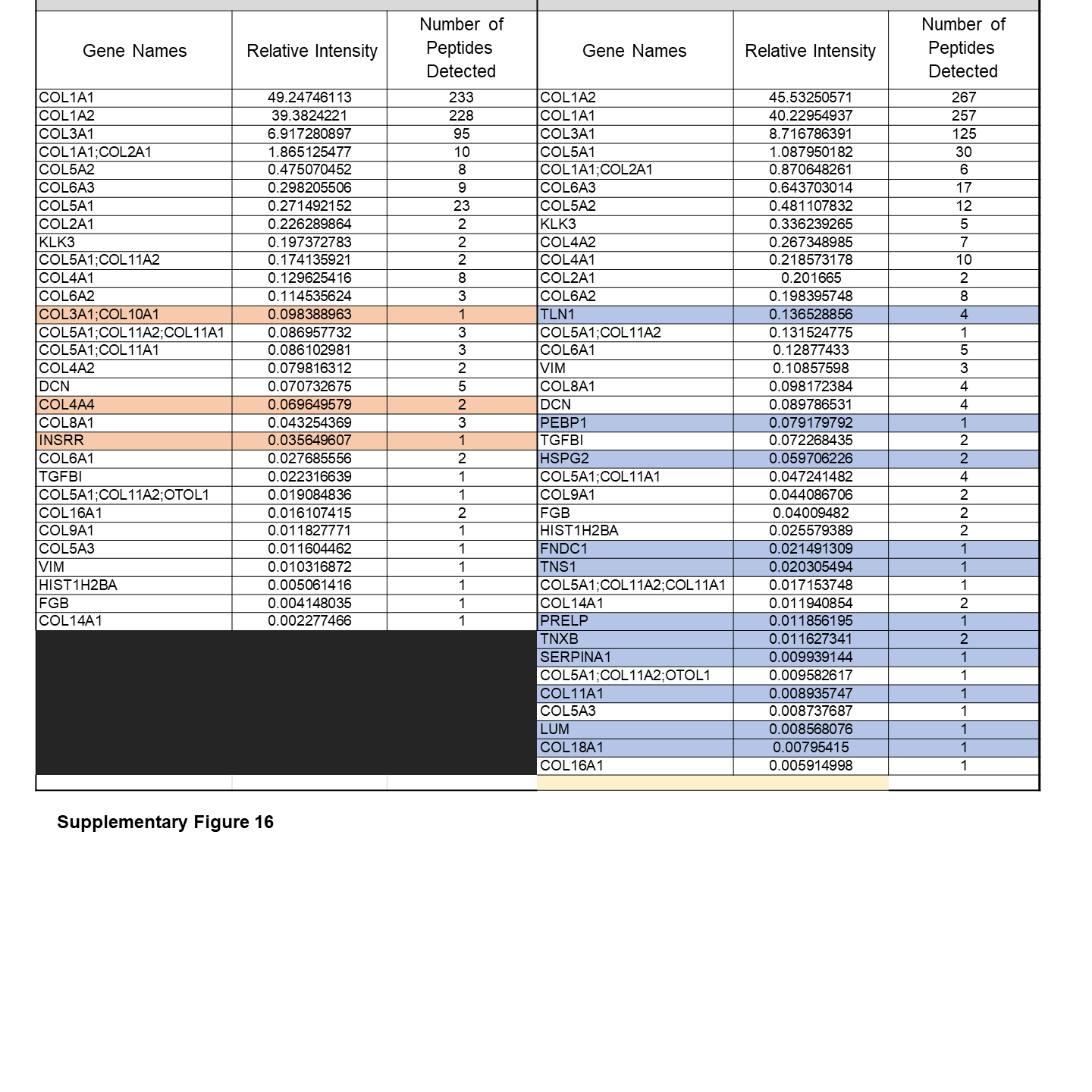

Supplement: Figure S16 — Table of all detected ECM peptides following LCMS analysis [file crc-24-0152_figure_s16_suppsf16.png]

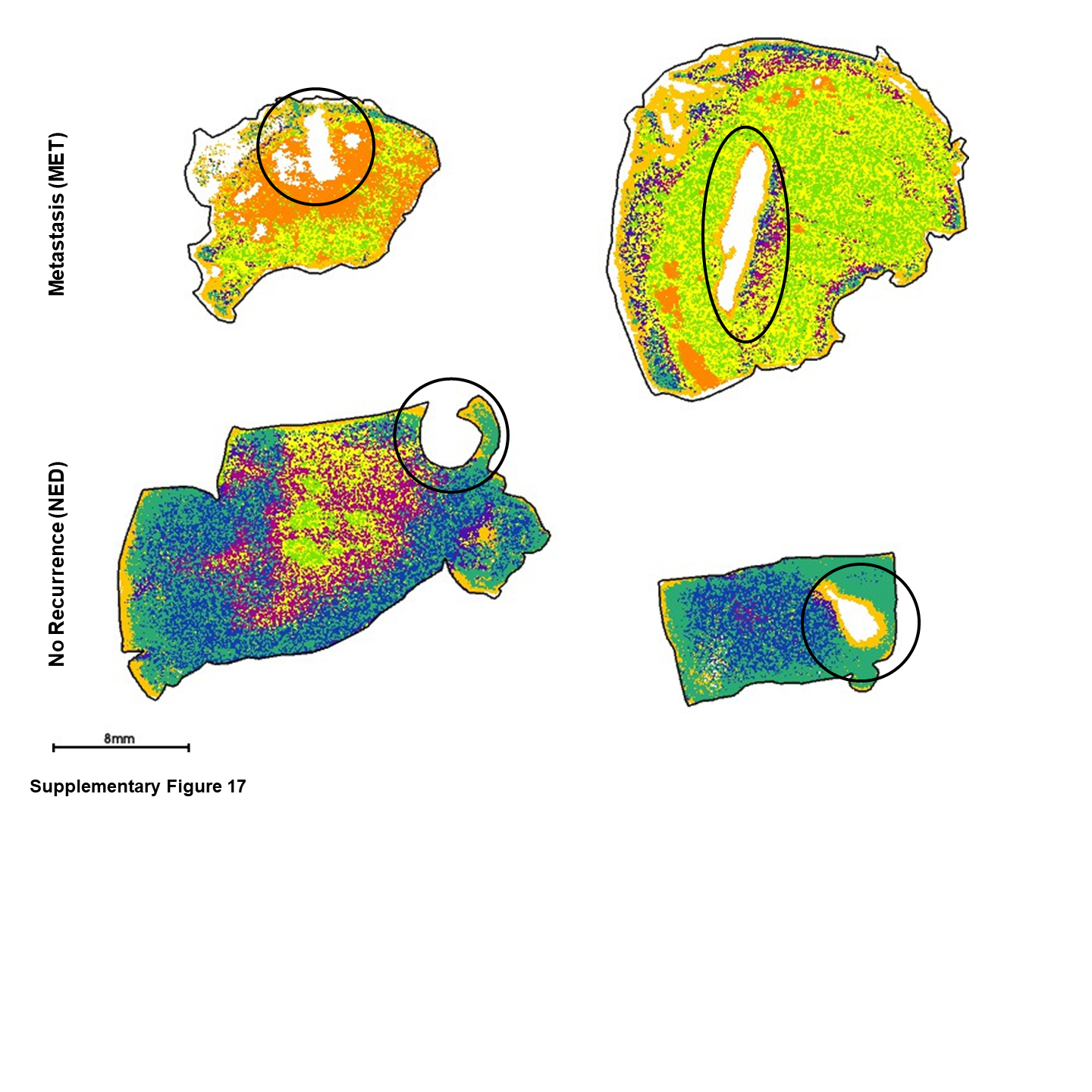

Supplement: Figure S17 — Segmentation analysis of data obtained from full tissue COLaseIII digest [file crc-24-0152_figure_s17_suppsf17.png]
