# Supplementary material for: Determining the N-Glycan and Collagen/Extracellular Matrix Protein Compositions in a Novel Outcome Cohort of Prostate Cancer Tissue Microarrays Using MALDI-MSI
Source: Cancer Res Commun. 2024 Nov 27;4(11):3036–48. doi: 10.1158/2767-9764.CRC-24-0152 (PMC11600299; doi:10.1158/2767-9764.CRC-24-0152)
Supplement: Supplementary Data Figure Legends — Figure Legends for Supplementary Data [file crc-24-0152_supplementary_data_figure_legends_suppflsd.docx]

**Supplemental Figure 1**: Hematoxylin and Eosin (H&E) stained TMA images. Images from the metastasis (MET) cohort shown in the top row and images from the no recurrence (NED) shown in the bottom.

**Supplemental Figure 2**: MALDI-IMS data of complete no recurrence (NED) cohort (top) and metastasis (MET) cohort (bottom) with corresponding m/z values and N-glycan structures shown to the right of each image, collected after PNGaseF digest.

**Supplemental Figure 3**: Classifications of N-glycans based on their structural characteristics. Each N-glycan was placed into at least one category based on their structural characteristics. There is overlap between the categories and most N-glycans can have more than one classification. These classifications determined grouping of peak intensities for statistical analysis. Five basic structures of N-glycans are pauci mannose, high mannose, complex, hybrid, and biantennary. Complex glycans can be separated into additional categories based on structural characteristics.

**Supplemental Figure 4**: ROC curves for specific N-glycans that were found to be capable of distinguishing between the “No Recurrence (NED)” cohort and the “Metastasis (MET)” cohort with corresponding AUROC values, p-values, and N-glycan structures shown. Data collected following PNGaseF digestion.

**Supplemental Figure 5**: Imaging data from EndoF3 enzyme digestion of Metastasis (MET) cohort and No Recurrence (NED) cohort and following PNGaseF digestion. Structures for each detected N-glycan shown in the bottom right corner of each image.

**Supplemental Figure 6**: MALDI-IMS data of complete metastasis (MET) cohort (top) and no recurrence (NED) cohort (bottom) with corresponding m/z values and N-glycan structures shown to the right of each image, collected after EndoF3 digest (right) digest and following PNGaseF digest (left).

**Supplemental Figure 7**: ROC curves for specific N-glycans that were found to be capable of distinguishing between the “No Recurrence (NED)” cohort and the “Metastasis (MET)” cohort with corresponding AUROC values, p-values, and N-glycan structures shown. Data collected following EndoF3 digestion (top) and following PNGaseF digestion (bottom).

**Supplemental Figure 8**: Segmentation analysis (Bisecting k-Means, weak denoising, Euclidean metric) of 8 tissue samples where TMAs were sampled from with sampling site circled in black. Top: Tissue samples from the “No Recurrence (NED)” cohort. Bottom: Tissue samples from the “Metastasis (MET)” cohort.

**Supplemental Figure 9**: MALDI-IMS data of complete no recurrence (NED) cohort (top) and metastasis (MET) cohort (bottom) with corresponding m/z values shown to the right of each image, collected after Collagenase III digest.

**Supplemental Figure 10**: **Supplemental Figure 9**: MALDI-IMS data of complete metastasis (MET) cohort (top) no recurrence (NED) cohort (bottom) with corresponding m/z values shown below each image, collected after Collagenase III digest.

**Supplemental Figure 11**: Analysis of collagen/ECM profile of NED vs MET cohort. A: Principal Component Analysis (PCA) plot created from 294 detected peptides. B: Partial Least Squares Discriminant Analysis (PLS-DA) plot and corresponding importance features plot for 294 detected peptides.

**Supplemental Figure 12**: Annotated spectra for collagen alpha-1(I) chain peptide corresponding to 1426.6600 m/z with annotated sequence and table detailing fragments. Corresponding image also shown.

**Supplemental Figure 13**: Annotated spectra for collagen alpha-1(I) chain peptide corresponding to 1178.5700 m/z with annotated sequence and table detailing fragments. Corresponding image also shown.

**Supplemental Figure 14**: Annotated spectra for collagen alpha-1(I) chain; collagen alpha-1(II) chain; collagen alpha 1(III) chain; Chondrocalcin peptide corresponding to 1301.6400 m/z with annotated sequence and table detailing fragments. Corresponding image also shown.

**Supplemental Figure 15**: Annotated spectra for collagen alpha-2(I) chain; collagen alpha-1(II) chain; chondrocalcin peptide corresponding to 890.425 m/z with annotated sequence and table detailing fragments. Corresponding image also shown.

**Supplemental Figure 16**: Complete list of all protein species and intensity and number of peptides detected in metastasis (MET) TMA cohort (left) and no recurrence (NED) cohort (right). Unique protein species highlighted in orange (MET) and blue (NED).

**Supplemental Figure 17**: Segmentation analysis (Bisecting k-Means, weak denoising, Euclidean metric) of 4 tissue samples where TMAs were sampled from with sampling site circled in black. Top: Tissue samples from the “Metastasis (MET)” cohort. Bottom: Tissue samples from the “No Recurrence (NED)” cohort.

**Supplemental Figure 18**: Table displaying glycan and collagen/ECM biomarker candidates that can be used to distinguish NED from MET PCa. AUC values shown in the leftmost column for each biomarker as well as for established diagnostic techniques (Gleason grade/stage NED v MET TMA, Decipher). Biomarker types to identify MET tissues are shown in the rightmost column.
